# Betaine inhibits Toll-like receptor 4 responses and restores intestinal microbiota in acute liver failure mice

**DOI:** 10.1038/s41598-020-78935-6

**Published:** 2020-12-14

**Authors:** Qian Chen, Yao Wang, Fangzhou Jiao, Chunxia Shi, Maohua Pei, Luwen Wang, Zuojiong Gong

**Affiliations:** Department of Infectious Diseases, Renmin Hospital of Wuhan University, Wuhan, 430060 China

**Keywords:** Applied microbiology, Medical research

## Abstract

Previous research has revealed that the gut microbiome has a marked impact on acute liver failure (ALF). Here, we evaluated the impact of betaine on the gut microbiota composition in an ALF animal model. The potential protective effect of betaine by regulating Toll-like receptor 4 (TLR4) responses was explored as well. Both mouse and cell experiments included normal, model, and betaine groups. The rat small intestinal cell line IEC-18 was used for in vitro experiments. Betaine ameliorated the small intestine tissue and IEC-18 cell damage in the model group by reducing the high expression of TLR4 and MyD88. Furthermore, the intestinal permeability in the model group was improved by enhancing the expression of the (ZO)-1 and occludin tight junction proteins. There were 509 operational taxonomic units (OTUs) that were identified in mouse fecal samples, including 156 core microbiome taxa. Betaine significantly improved the microbial communities, depleted the gut microbiota constituents *Coriobacteriaceae, Lachnospiraceae, Enterorhabdus* and *Coriobacteriales* and markedly enriched the taxa *Bacteroidaceae, Bacteroides, Parabacteroides* and *Prevotella* in the model group*.* Betaine effectively improved intestinal injury in ALF by inhibiting the TLR4/MyD88 signaling pathway, improving the intestinal mucosal barrier and maintaining the gut microbiota composition.

Acute liver failure (ALF), characterized by massive liver necrosis associated with severe impairment of hepatic function, is an intractable and high-mortality disease in clinical practice^1^. Regarding the pathogenesis of liver failure, the majority of scholars support the theory of the ‘two-hit hypothesis’. Hepatitis virus, ethanol, and hepatotoxicants always lead to liver injury directly, which is regarded as the primary hit. Intestinal endotoxemia, as the secondary cause of liver injury, also plays an important role in the occurrence and development of ALF^2^. In addition, liver failure patients are susceptible to mixed endotoxemia. This phenomenon is due mainly to the damaged intestinal mucosal barrier, which leads to a significant quantity of endotoxin (lipopolysaccharide, LPS) produced by the overgrowth of gram-negative bacteria^3^.

The integrated intestinal wall barrier consists mainly of the intestinal mucosal epithelium, tight junctions and intrinsic membrane under the epithelium^4^. The tight junction between epithelial cells contains the transmembrane proteins occludin, claudins, junctional adhesion molecules, and cytoplasm protein (ZO)-1. They are the most imperative part of the intestinal wall barrier^5^. Intestinal mucosal permeability is increased when these junctions are altered^6^. There is a close relationship between weakened intestinal mucosal immune function and mucosal mechanical barrier damage by bacteria. Both contribute to translocation of endotoxin and excessive intestinal pathogens^7^. Endotoxin is an LPS component that is recognized as an outer membrane component in gram-negative bacteria. Toll-like receptors (TLRs) are pattern recognition receptors (PRRs). They play a valuable role in the intestinal mucosal immune system by mediating signal transduction. In particular, TLR4-mediated recognition of LPS occurs via the formation of two copies of the TLR4–MD2–LPS complex as a receptor multimer. Then, the complex recruits MyD88 (MyD88-dependent pathway) and TIR domain–containing adaptors (TIRAP, Mal). Once the MyD88-dependent pathway is activated, downstream signaling molecules are further activated, which leads to the release of many inflammatory mediators, including TNF-α, IL-1β and IL-18^8–10^. A previous study indicated that the transcription of TLR4 is enhanced in ALF mice^11^.

Increasing evidence has shown a close correlation between the gut microbiome and liver diseases^12^. The liver conducts immune surveillance for multifarious pathogens from the gut, as well as influences intestinal mucosal immunity. The intestinal microbiome also affects liver function^13^. The mutual effect between the gut and liver is regarded as the “gut-liver axis”. The integrity of the intestinal epithelial barrier has been demonstrated to be regulated by the gut microbiota^14^. A compromised intestinal mucosal barrier contributes to bacterial translocation into the portal vein and then dissemination to the liver. This process leads to additional inflammation, liver cell apoptosis, and rapid progression to multiple organ failure^15^. The abundance of the pathogenic genus *Proteus* is increased in ALF rats, and *Coriobacteriaceae*, *Bacteroidales* and *Allobaculum* are markedly depleted in ALF rats^16^. Accumulating evidence suggests that the gut microbiota is involved in this process. Moreover, the abundances of *Bacteroidetes*, *Ruminococcaceae*, *Porphyromonadaceae* and *Lachnospiraceae* are decreased in fecal microbial communities in acute-on-chronic liver failure (ACLF) patients compared to their levels before disease onset^17^. In addition, the levels of *Firmicutes* are increased in ACLF patient feces. The regulation of the intestinal microecology by microbial ecological agents has also been proposed as an emerging therapeutic strategy for liver failure^18^.

Betaine, as a vital human nutrient, exists in many tissues and organs and is especially abundant in the liver and kidney. It participates in the methyl cycle. It is often used as a feed additive to replace the biological function of methionine to reduce the cost of feedstuffs^19^. Many studies have shown that betaine has many pharmacological functions, such as antioxidant and anti-inflammatory effects^20,21^. A retrospective study suggested that higher betaine intake may be related to a lower risk of primary liver cancer^22^. There is a favorable relationship between the blood betaine concentration and the severity of nonalcoholic fatty liver disease (NAFLD) in community-based participants^23^. Our previous studies have also indicated that betaine inhibited TLR4 expression to effectively improve alcoholic liver disease and NAFLD^24,25^. However, the effects of betaine on ALF and related mechanisms are still unknown. In particular, the influence of betaine on the gut microbial ecosystem needs further study.

At present, high-throughput next-generation sequencing techniques have been applied. They assist us in identifying the overall structure of the complex gut microbial ecosystem at the OTU level. In this study, we employed D-Gal/LPS in vivo and LPS in vitro to establish internal and external intestinal epithelial barrier disruption models of ALF, respectively. Betaine was employed as an intervention agent. The relationship between the expression of key molecules in the intestinal TLR4-mediated signaling pathway and intestinal tight junction proteins was examined. The gut microbial ecosystem was also studied to explore the potential therapeutic effect of betaine in acute liver failure.

## Materials and methods

### Reagents

Fetal bovine serum (FBS) and DMEM basic were purchased from Gibco (NY, USA). Betaine hydrochloride (purity of 99%) was purchased from Juhua Group Co. (Zhejiang, China). Lipopolysaccharide (LPS, purity of 99%) and D-galactosamine (D-Gal, purity of 98%) were purchased from Sigma (St. Louis, USA). Antibodies against TLR4, (ZO)-1 and GAPDH were purchased from Proteintech (Hubei, China). Rabbit anti-rat/mice occludin and MyD88 were purchased from Cell Signaling Technology (Boston, USA). The Goat anti-rabbit fluorescent secondary antibody (IRDye800) was purchased from LI-COR Biosciences, Inc. (Lincoln, USA).

### Cell culture

The rat small intestinal cell line IEC-18 was grown in DMEM medium with 10% FBS in an incubator at 37 °C, 5% CO_2_, and saturated humidity. LPS (1 μg/ml) was applied to cells in the model group, and low dose (3.4 mM), medium dose (5.1 mM) and high dose (6.8 mM) betaine groups were established. IEC-18 cells were seeded in 6-well plates. After 12 h, betaine (3.4 mM, 5.1 mM or 6.8 mM) was added to the different dose betaine groups. In addition, betaine (3.4 mM, 5.1 mM or 6.8 mM) only group was also set. All cells were harvested after models were incubated for 24 h.

### Transepithelial electrical resistance (TEER) measurement

IEC-18 cells were seeded at a 2.5 × 10^5^ cells/ml single-cell suspension. Then, 1.5 ml of DMEM complete medium was added to the lower cell chambers, and 1 ml of the cell suspension was sequentially added to the upper cell chambers. The media in the upper and lower chambers in the betaine group were replaced by complete medium containing betaine. After 2 h, LPS (1 μg/ml) was added to the model group and betaine group. After administrating LPS, they were incubated for 24 h. The next experimental steps were performed as described in our previous report^26^. And then the calibrated Millipore Millicell ERS-2 cell resistance meter was used to detect resistance value. The measured resistance value was multiplied by the area of the filter to obtain an absolute value of TEER, expressed as Ωcm^2^. And the TEER values were measured as follows: TEER = (measured resistance value − blank value) × single cell layer surface area (cm^2^).

### Animal groups

Eighteen male specific pathogen-free (SPF) mice weighing 20 ± 2 g were purchased from the Experimental Animal Center of Wuhan University. Animal experiments were approved by the Animal Care and Use Committee of Renmin Hospital of Wuhan University. The protocol was in accordance with the Guide for the Care of Laboratory Animals published by the US National Institutes of Health (NIH publication no. 85-23, revised 1996). Experimental animals were kept at an appropriate temperature (22 ± 2 °C) with a 12 h light/dark cycle and were allowed free access to food and water. After acclimation for 1 week, they were randomly divided into three groups: the normal, model, and betaine groups. D-Gal (400 mg/kg) and LPS (100 μg/kg) were administered by intraperitoneal injection in ALF model animals. Betaine (800 mg/kg per day) was administered intragastrically in the betaine groups one week before the ALF model was established. The other mice were administrated intragastrically the same amount of saline. Experimental animals were sacrificed 24 h after model and betaine group mice were given D-Gal/LPS.

### Assessment of liver function and inflammatory mediators

Blood samples were collected after mouse euthanasia. A Hitachi Automatic Analyzer (Hitachi, Inc., Japan) was applied to detect serum alanine aminotransferase (ALT), aspartate aminotransferase (AST) and total bilirubin (TBIL) levels in mice. The levels of the serum inflammatory cytokines tumor necrosis factor α (TNF-α), interleukin-1β (IL-1β) and IL-18 were determined by ELISA kits (eBioscience, CA, USA). The level of TNF-α, IL-1β and IL-18 in liver and small tissue were detected by Quantitative real-time PCR.

### Histological examinations

Liver and small intestine specimens were fixed with 10% formaldehyde, embedded in paraffin, and cut into sections with 5 μm thickness. Then, they were stained with hematoxylin–eosin (HE) for pathological studies under a BX 51 light microscope (Olympus, Japan).

### Quantitative real-time PCR

Total RNA was extracted from small intestine tissue and IEC-18 cells using TRIzol reagent based on the manufacturer’s procedure. qRT-PCR was conducted with a SYBR Green PCR Kit (Takara Bio, Inc., Otsu, Japan). All primers (sequences in Table 1) were constructed by Tsingke (Wuhan, China). In this study, β-actin was selected as the housekeeping gene.

**Table 1:** The primer sequences for RT-PCR.

Genes	Forward (5′–3′)	Reverse (5′–3′)
TNF-α (mouse)	CGTCAGCCGATTTGCTATCT	CGGACTCCGCAAAGTCTAAG
IL-1β (mouse)	TCAGGCAGGCAGTATCACTC	AGCTCATATGGGTCCGACAG
IL-18 (mouse)	GACAGCCTGTGTTCGAGGATATG	TGTTCTTACAGGAGAGGGTAGAC
TLR4 (rat)	TACAGTTCGTCATGCTTTCTC	ATTAGGAAGTACCTCTATGCAG
TLR4 (mouse)	AGCTTCTCCAATTTTTCAGAACTTC	TGAGAGGTGGTGTAAGCCATGC
MyD88 (rat)	AGGACAAACGAAGGAACTTTT	GCCGATAGTCTGTCTGTTCTAGT
MyD88 (mouse)	ACCTGTGTCTGGTCCATTGCCA	GCTGAGTGCAAACTTGGTCTGG
(ZO)-1 (rat)	GCTCACCAGGGTCAAAATGT	GGCTTAAAGCTGGCAGTGTC
(ZO)-1 (mouse)	GTTGGTACGGTGCCCTGAAAGA	GCTGACAGGTAGGACAGACGAT
Occludin (rat)	TTACGGCTATGGAGGGTACAC	GACGCTGGTAACAAAGATCAC
Occludin (mouse)	TGGCAAGCGATCATACCCAGAG	CTGCCTGAAGTCATCCACACTC
β-Actin (rat)	GTCGTACCACTGGCATTGTG	CTCTCAGCTGTGTGTGTGAA
β-Actin (mouse)	CTCTCAGCTGTGTGTGTGAA	TGCTGGAAGGTGGACAGTGAGG

### Western blotting

The IEC-18 cells and small intestine specimen extracts were subjected to 10% sodium dodecyl sulfate–polyacrylamide gel electrophoresis (SDS-PAGE). Then, they were transferred to a Protran nitrocellulose membrane. The membrane was sequentially incubated with a primary antibody at 4 °C overnight and secondary antibody for 1 h. Finally, an Odyssey infrared imaging system (LI-COR Co.) was utilized to detect the protein levels. The protein levels of TLR4, MyD88, (ZO)-1, and occludin were normalized to that of GAPDH for each sample. The dilution ratio of all primary antibodies was 1:1000, and the dilution of secondary antibodies was 1:10,000. Incubating different primary antibodies after cutting on a whole membrane results in the absence of images of adequate length. But this experiment ensures that the gray value determination of each group of proteins on the same band is detected within the same size range.

### Intestinal permeability

The everted sac method was employed to evaluate the small intestinal mucosal barrier function as previously described^27^. Segments were everted in Krebs buffer which was ice-cold and pH was 7.4, gently distended by injecting 1.5 ml Krebs. They were suspended in the organ bath for 30 min and maintained at 37 °C. The organ bath consisted of 500-ml Krebs with added FITC labeled dextran 4000 (FD4, 10 mg/ml), continuously bubbled with a gas mixture containing 95% O_2_ and 5% CO_2_. And then centrifuged at 1000*g* at 4 °C for 5 min. FD4 concentration was measured at an excitation wavelength of 492 nm and an emission wavelength of 515 nm with PerkinElmer LS-50 fluorescence spectrophotometer (PerkinElmer Inc., Waltham, MA). Intestinal permeability was presented as FD4 concentration divided by the area of gut sac.

### DNA extraction and 16S rRNA gene sequencing

Within 1 h before all animals were sacrificed, fecal samples were collected. They were frozen at − 80 °C immediately. DNA was extracted using a QIAGEN extraction kit (Universal Biotech Company, Shanghai, China). Total DNA quality was assessed by using a Thermo Qubit.

### Sequencing library construction

The V3-4 region of the 16S rRNA gene was amplified using custom barcoded primers and sequenced as described previously using an Illumina MiSeq sequencer^28^. Briefly, the V3-4 domain of the 16S rRNA gene was amplified using the primers F (5′-CTACGGGNGGCWGCAG-3′) and R (5′-GACTACHVGGGTWTCTAAT-3′). Employing diluted genomic DNA as a template, PCR was performed utilizing Taq DNA Polymerase (Vazyme Biotech Company, Nanjing, China) to ensure the accuracy and efficiency of the amplification. Then, the PCR product library was examined by a Fragment Analyzer. After the library quality was deemed eligible, the corresponding ratios were mixed according to the volume required in each sample. The mixed library was subjected to gel purification (cutting range: 500–750 bp) using a QIAquick gel recovery kit (Universal Biotech Company, Shanghai, China). After purification, the Fragment Analyzer and an Applied Biosystems QuantStudio 6 real-time PCR instrument were used to examine and quantify the library. Sequencing was performed using an Illumina MiSeq PE300.

After performing quality control of the original data, Usearch software was applied to de-chimerize and cluster the data. For Usearch clustering, reads were first sorted according to the abundance from large to small, and the standard clustering of 97% similarity was obtained. Each operational taxonomic unit (OTU) was considered to represent a species. Next, the reads of each sample were randomly leveled, and the corresponding OTU sequence was extracted. Then, we used QIIME software to generate a dilution curve of the alpha diversity index, selected reasonable sampling parameters according to the dilution curve, and analyzed the obtained OTUs. A read was extracted as a representative sequence from the OTU. Next, according to the 16S rRNA database, the representative sequence was applied to classify each OTU by using the RDP method. After categorization, an OTU abundance table was obtained in line with the number of sequences belonging to each OTU. Finally, subsequent analysis was performed according to the OTU abundance table^29^.

### Statistical analysis

All data are presented as the mean ± standard deviation. Statistical significance was determined using the analysis of variance (ANOVA) method, followed by Bonferroni's post hoc test. A *P* value < 0.05 was considered statistically significant. Calculations were performed with SPSS 16.0. Alpha diversity was used to analyze the complexity of species diversity for a single sample. Beta diversity analysis was applied to assess differences in species complexity among samples. LEfSe was used by linear discriminant analysis (LDA) to evaluate the influence of each species abundance to determine significant communities or species in sample partitioning.

## Results

### Betaine ameliorated liver and small intestine injury in ALF mice

**Figure 1:**
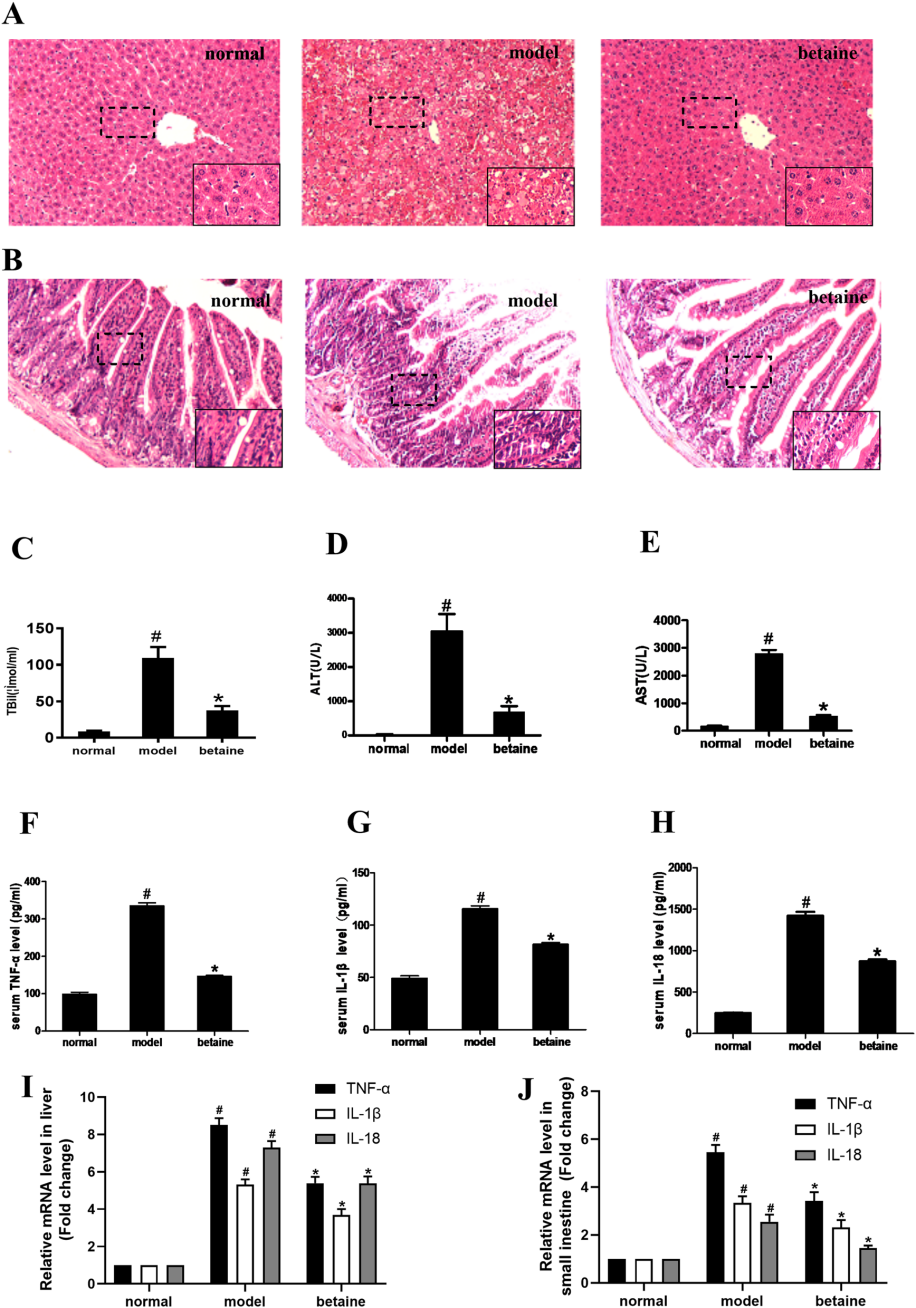
Effect of betaine on liver and small intestine tissue pathological changes and serum biochemical indicators in ALF mice. (**A**) The liver tissues were stained with HE (× 200). (**B**) The small intestine tissues were stained with HE (× 200). (**C**–**E**) The serum levels of ALT, AST, and TBIL in different animal groups. ^*#*^*P* < 0.05, compared with the normal group; *P < 0.05, compared with the model group. (**F**–**H**) The serum levels of TNF-α, IL-1β and IL-18 in each group. (**I**,**J**) The relative mRNA levels of TNF-α, IL-1β and IL-18 in liver and small intestine tissue. ^*#*^*P* < 0.05, compared with the normal group; *P < 0.05, compared with the model group.

**Figure 2:**
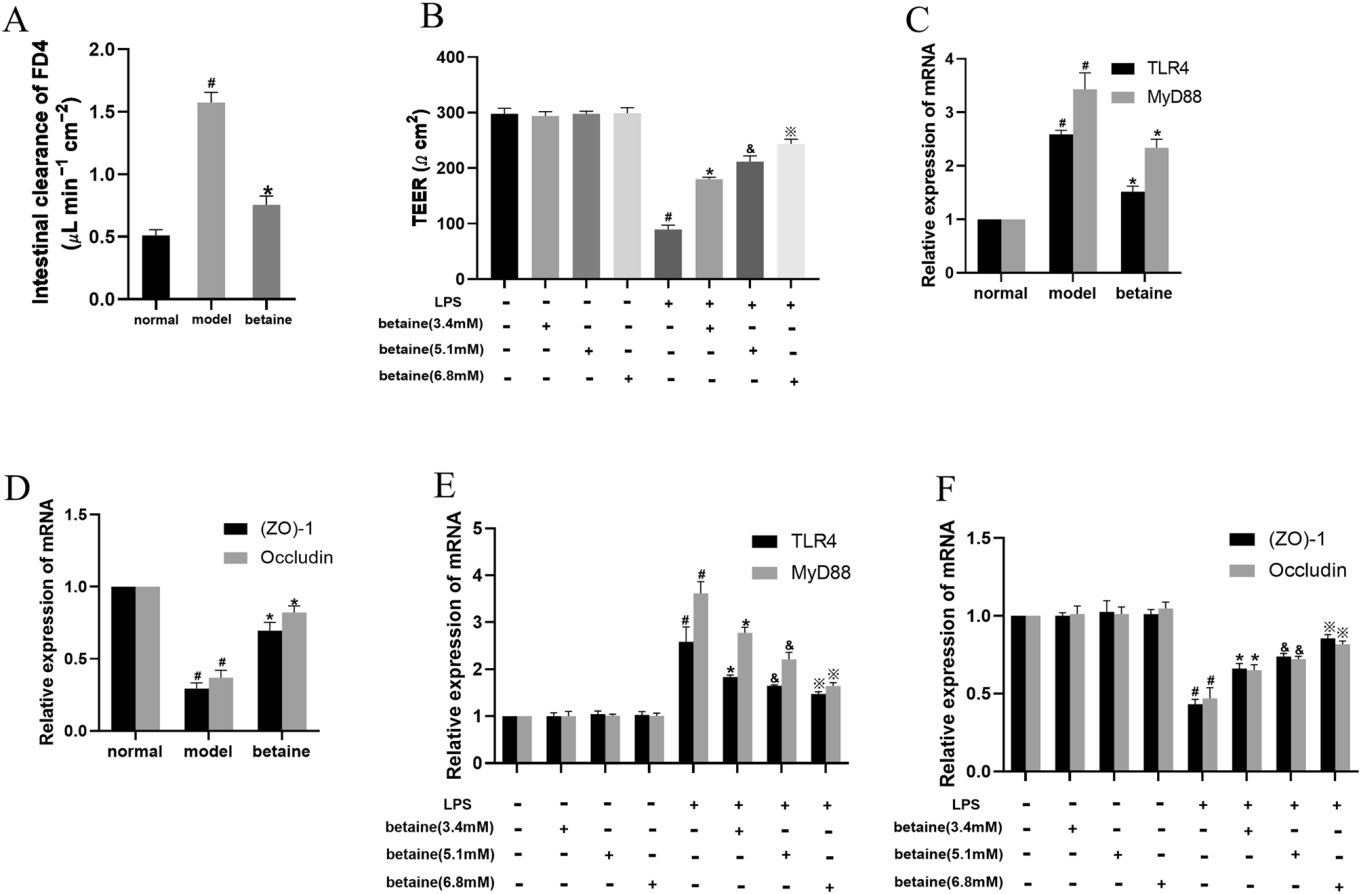
Effect of betaine on the TLR4/MyD88 pathway, (ZO)-1 and occludin mRNA levels and intestinal permeability in ALF mice and LPS-stimulated IEC-18 cells. (**A**) Intestinal permeability in each animal group. (**B**) The TEER value in different cell groups. (**C**,**D**) The mRNA levels of TLR4, MyD88, (ZO)-1 and occludin in different animal groups. (**E**,**F**) The mRNA levels of TLR4, MyD88, (ZO)-1 and occludin in different cell groups. ^*#*^*P* < 0.05, compared with the normal group; *P < 0.05, compared with the model group; ^&^*P* < 0.05, compared with the low-dose betaine group; ^*※*^*P* < 0.05, compared with the medium-dose betaine group.

Pathological changes in mouse liver tissue and serum biochemical markers were assessed. The structure of liver lobules and the arrangement of liver cells were clear and orderly in the normal group. There was also no necrosis of hepatocytes or infiltration of inflammatory cells in the normal group. However, in the model group, the liver lobular structure was destroyed, and a larger amount of hepatocyte necrosis was observed. Compared with model group, the degrees of hepatocyte necrosis were lessened in the betaine group (Fig. 1A). Serum ALT, AST and TBIL levels in the model animals were significantly increased compared with those in the normal group animals (*P* < 0.05). Nevertheless, the ALT, AST and TBIL levels were much lower in the betaine groups than in the model group (*P* < 0.05) (Fig. 1C–E). As shown in Fig. 1B, histological analysis indicated that the lamina propria and villi of the model group were denuded, the epithelial layer of the small intestine was exfoliated, the height of the small intestine was reduced. In contrast, betaine conserved almost normal architecture in the small intestine. Compared with those in the normal group, the serum levels and liver and small intestine mRNA levels of TNF-α, IL-1β and IL-18 were greatly increased in the model group. Betaine significantly decreased the levels of TNF-α, IL-1β and IL-18 in the model group (*P* < 0.05) (Fig. 1F–J). Moreover, the intestinal permeability remarkably increased in the model group compared with that in the normal group (*P* < 0.05). Betaine dramatically improved intestinal permeability in the model animals (*P* < 0.05) (Fig. 2A).

### Betaine inhibited the TLR4/MyD88 pathway and improved the mRNA and protein expression of (ZO)-1 and occludin in ALF mice

**Figure 3:**
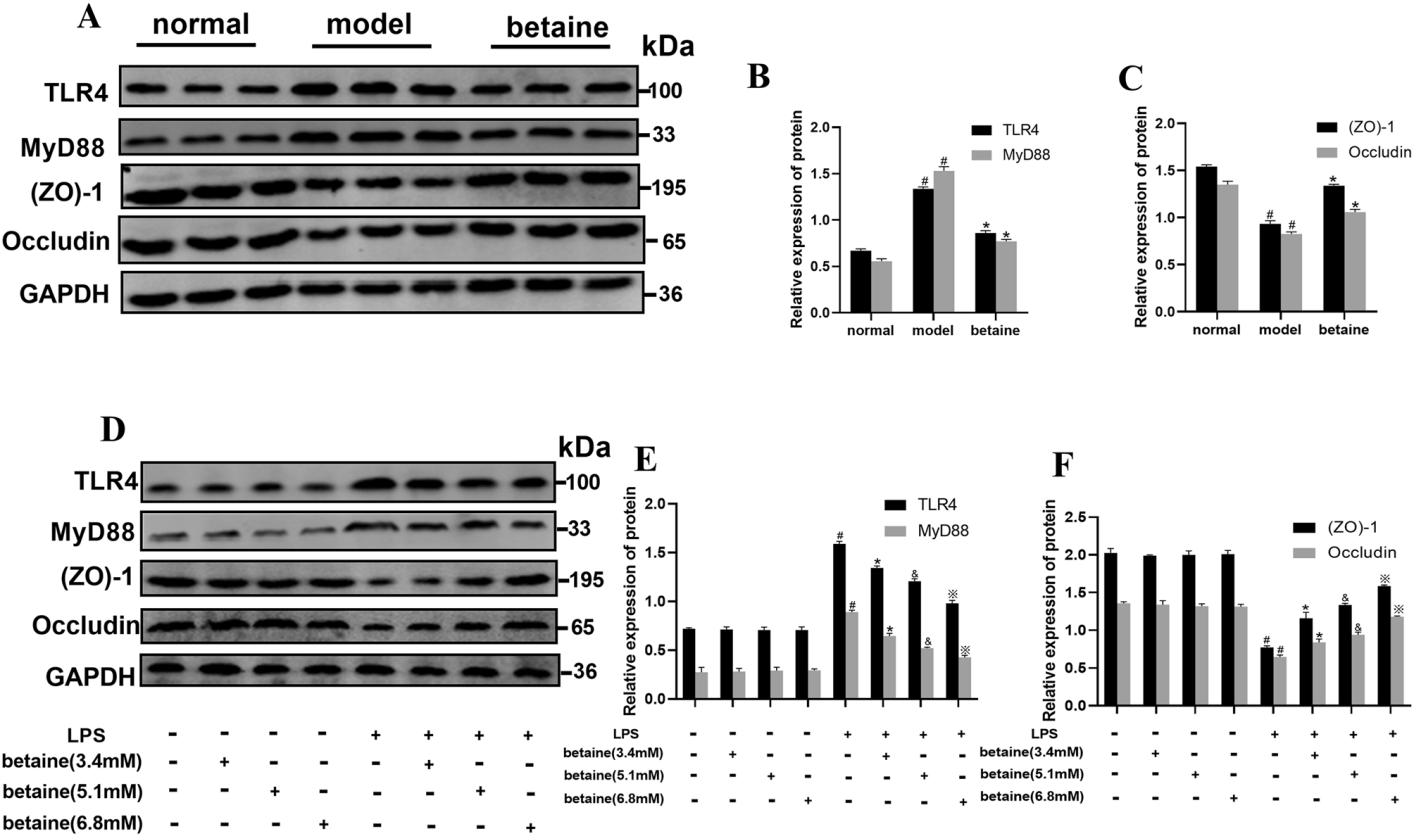
Effect of betaine on the TLR4/MyD88 pathway, (ZO)-1 and occludin protein levels in ALF mice and IEC-18 cell induced with LPS. (**A**–**C**) The protein levels of TLR4, MyD88, (ZO)-1 and occludin in different animal groups. (**D**–**F**) The protein levels of TLR4, MyD88, (ZO)-1 and occludin in different cell groups. ^*#*^*P* < 0.05, compared with the normal group; *P < 0.05, compared with the model group; ^&^*P* < 0.05, compared with the low-dose betaine group; ^*※*^*P* < 0.05, compared with the medium-dose betaine group.

The effect of betaine by regulating the TLR4/MyD88 pathway in ALF model mice was assessed. Compared with those in the normal group, the mRNA and protein level of the TLR4 and MyD88 were obviously increased in the model group (*P* < 0.05). The mRNA and protein level of TLR4 and MyD88 were significantly decreased in the betaine group compared with those in the model group (Figs. 2C, 3A, B). Furthermore, the results showed that the mRNA levels of (ZO)-1 and occludin were both distinctly decreased in the model group compared with those in the normal group (*P* < 0.05). Betaine significantly elevated the expression of (ZO)-1 and occludin in the model animals (*P* < 0.05) (Figs. 2D, 3A,C).

### Betaine suppressed the TLR4/MyD88 pathway and enhanced the levels of (ZO)-1 and occludin in IEC-18 cells stimulated by LPS

Cell experiments were performed to verify the protective effect of betaine in LPS-stimulated IEC-18 intestinal epithelial cells. The results showed that the mRNA levels of TLR4 and MyD88 were prominently increased in the model group compared with those in the normal group (*P* < 0.05). Compared with those for the model treatment, the mRNA and protein level of TLR4 and MyD88 were dramatically suppressed by betaine administration (*P* < 0.05). The TLR4 and MyD88 mRNA and protein level were also significantly different among the high-, medium- and low-dose betaine groups (Figs. 2E, Fig. 3D,E, P < 0.05). As shown in Figs. 2F, 3D,F, compared with those in the normal group, the mRNA and protein expression level of (ZO)-1 and occludin were markedly reduced in the model group (*P* < 0.05). The mRNA and protein expression of (ZO)-1 and occludin were significantly improved in the betaine group compared with those in the model group (*P* < 0.05). Moreover, the mRNA and protein level of (ZO)-1 and occludin in the medium-dose betaine group were elevated compared with those in the low-dose betaine group and were the highest in the high-dose betaine group (*P* < 0.05). This increased expression also showed dose dependence. But there was no statistical significance between normal group and only betaine group (high-, medium- and low-dose) in protein and mRNA level of TLR4, MyD88, (ZO)-1 and occludin.

### Betaine elevated the TEER value in LPS-stimulated IEC-18 cells

As shown in Fig. 2B, the TEER value was greatly decreased in the model group compared with that in the normal group (*P* < 0.05). The TEER value was significantly elevated after betaine treatment in the model group (*P* < 0.05). In addition, there were significant differences among the high-, medium- and low-dose betaine groups (*P* < 0.05). And there was no statistical significance between normal group and only betaine group (high, medium and low-dose) in TEER value.

### OTU analysis

**Figure 4:**
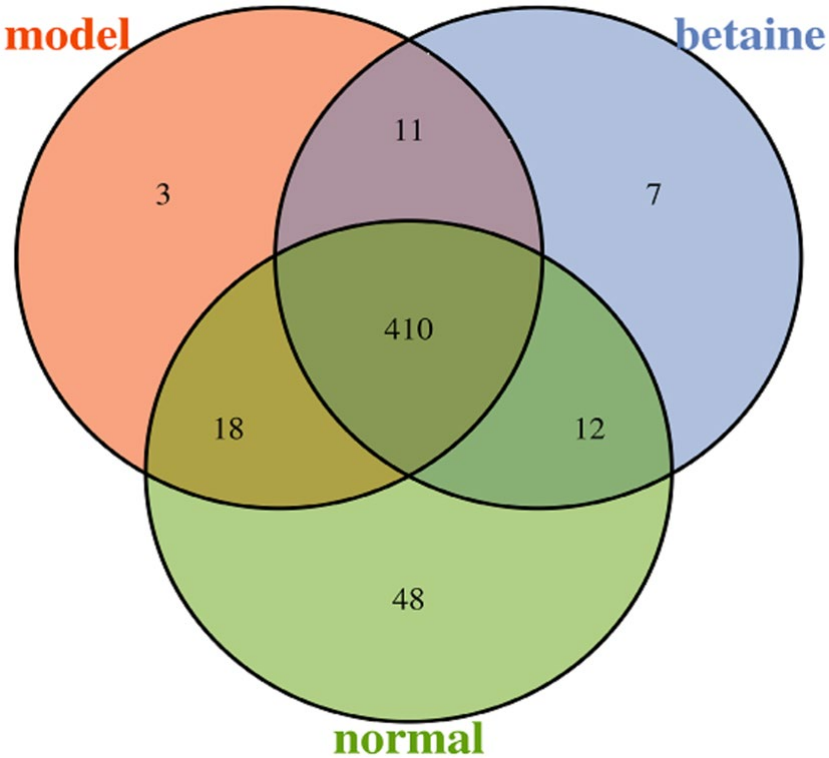
OTU analysis. OTU Venn diagram showing different color patterns representing different groups, and the number of overlaps between different color patterns was the number of OTUs shared between the two groups.

In our experiments, 16S rRNA gene sequence analysis was used to explore the potential therapeutic microecological mechanisms of betaine in ALF mice. The OTU network analysis for fecal samples of mice provided certain core OTUs and a universal microbial composition among each group. A total of 509 OTUs were identified from 15 fecal samples (Supplementary files Table S1). As shown in Fig. 4, the normal group contained the maximum OTUs, but the betaine and model groups possessed a similar number of OTUs. The core microbiome that was present in each fecal sample could be found based on the shared OTUs of each sample and the species represented by the OTUs. There were 156 core microbiome constituents in fecal samples (Supplementary files Table S2).

### Alpha diversity analysis

Alpha diversity contains the observed species index, the Chao1 index, the Shannon index and the Simpson index. The observed species index indicates the actual number of OTUs observed, and the Chao1 index is performed to calculate the total number of OTUs contained in a sample. Both of them indicate the species richness of the sample. The Simpson index and Shannon index are applied to evaluate species diversity. As shown in Table 2, the species richness and diversity of the microbiota among the three groups did not have notable differences. However, the fecal bacteria in the normal group had relatively higher levels of species richness and diversity than those in the other groups.

**Table 2:** Alpha diversity analysis.

Sample name	Observed species	Chao1	Shannon	Simpson
p value	0.1	0.22	0.09	0.09
Mean (normal)	383	411.79	6.64	0.98
Mean (model)	364.2	397.32	6.38	0.97
Mean (betaine)	337.8	378.8	6.32	0.98

### Beta diversity analysis

**Figure 5:**
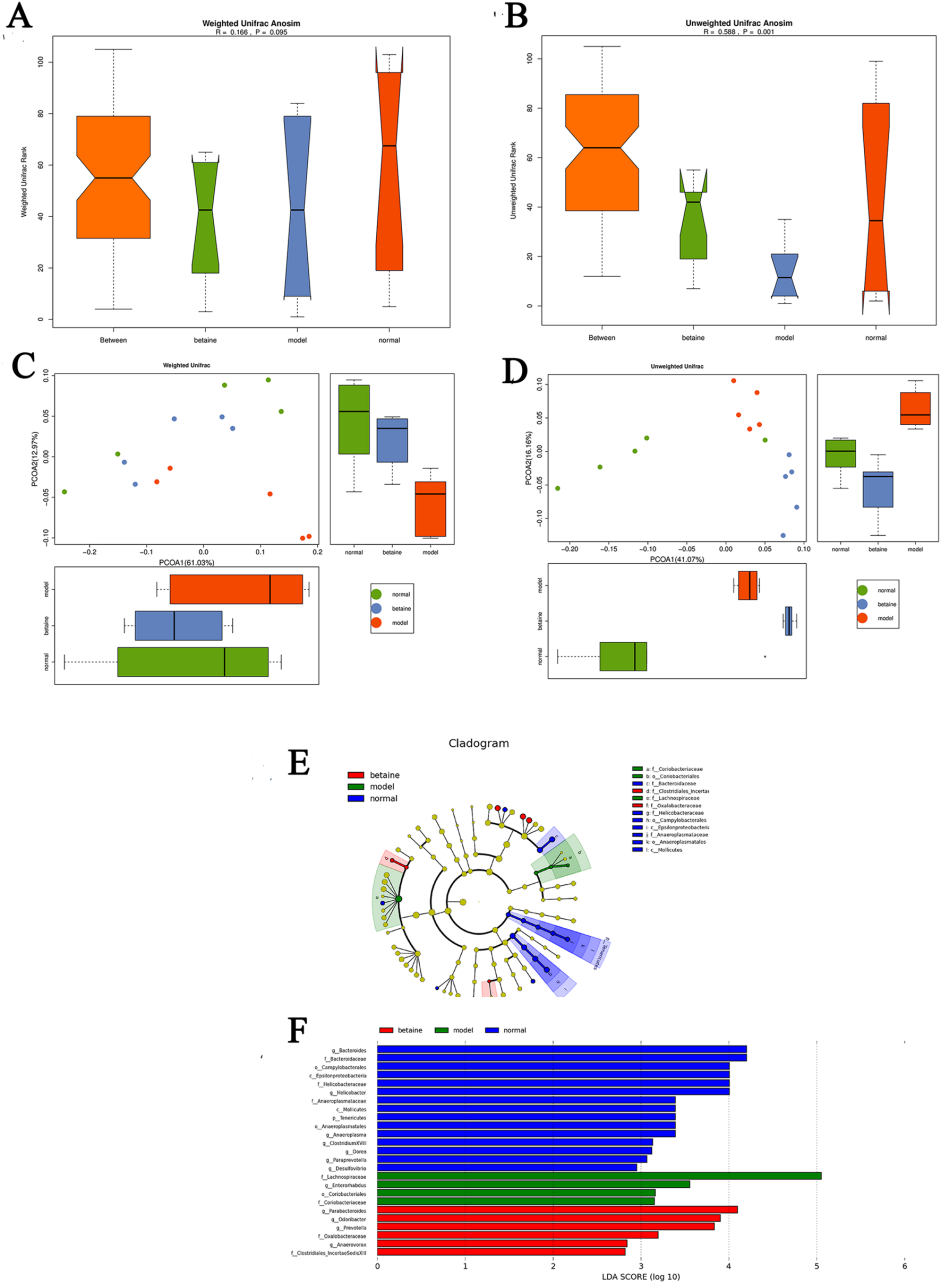
Beta diversity, including ANOSIM and PCoA. (**A**,**B**) Weighted UniFrac ANOSIM and unweighted UniFrac ANOSIM; (**C**,**D**) weighted UniFrac PCoA and unweighted PCoA. (**E**,**F**) LEfSe showed a cluster tree (**E**), and a histogram (**F**) representing the gut bacteria, which were of important biological significance in each group.

Different from alpha diversity analysis, beta diversity analysis is appropriate to distinguish the differences in species diversity among a pair of samples. UniFrac compares species community differences using phylogenetic evolution information. The results can serve as an index to detect beta diversity, which has taken into account the evolutionary distance between species. UniFrac results are classified into weighted UniFrac and unweighted UniFrac. Weighted UniFrac considers the abundance of sequences, and unweighted UniFrac does not. Beta diversity analyses include ANOSIM and principal coordinates analysis (PCoA). ANOSIM is a nonparametric test used to detect whether the difference between two or more groups is significantly greater than the intragroup difference to judge whether the grouping is reasonable. As shown in Fig. 5A,B, ANOSIM using weighted UniFrac distances and unweighted UniFrac clustered samples. The data revealed that intermouse variations in the fecal microbiota were lower than the intragroup variations, which showed that the fecal microbiota of each group had better individual similarity. PCoA showed that the distances between two samples were close, which indicated that the species compositions of the two samples were similar. The results of PCoA showed that the microbial composition between the normal group and betaine group was more similar than that between the normal and model groups (Fig. 5C,D).

### LDA effect size (LEfSe)

**Figure 6:**
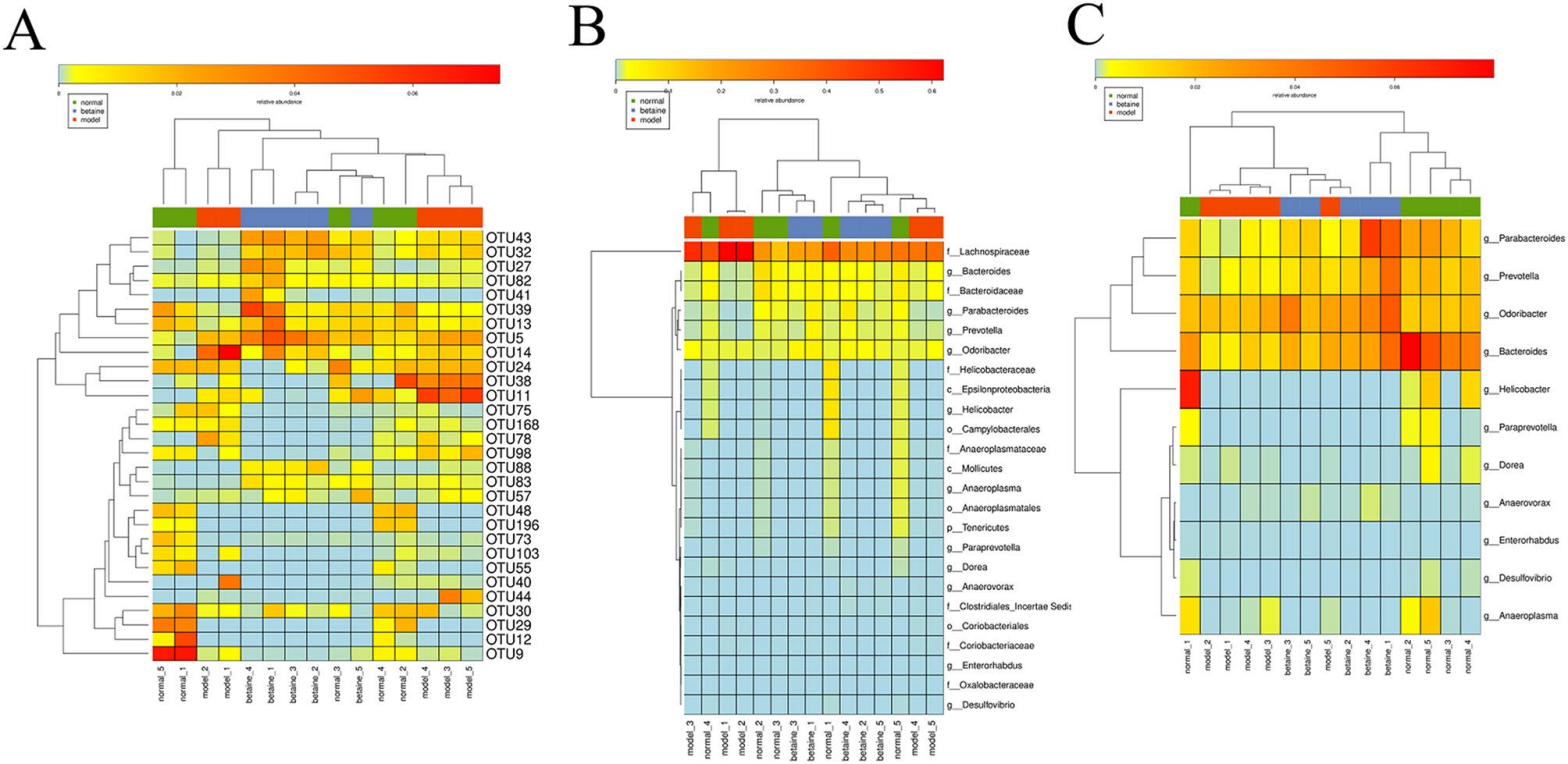
LEfSe showed three heatmaps. (**A**) The OTUs with a significant difference between different groups. (**B**) A total of 24 species with significant differences between groups. (**C**) There were 11 genera that were significantly different between groups.

LEfSe emphasizes statistical significance and biological relevance. As shown in Fig. 5E,F, a cluster tree displayed different colors representing different groups, and nodes of different colors represented corresponding important microorganisms in each group. However, the yellow node represents nonsignificant microbes. The 11 bacterial species in fecal samples, such as *g-Bacteroide*, *f-Bacteroidaceae*, *o-Campylobacterales* and *c-Epsilonproteobacteria,* had a significant effect on the normal group. *F-Lachnospiraceae*, *g-Enterorhabdus*, *o-Coriobacteriales* and *f-Coriobacteriaceae* contributed greatly in the model group. *G-Parabacteroides*, *g-Odoribacter*, *g-Prevotella*, *f-Oxalobacteraceae*, *g-Anaerovorax* and *f-Clostridiales-IncertaeSedisXIII* were of crucial importance in the betaine group. As shown in Fig. 6A and Supplementary files Table S3, there were a total of 143 OTUs that had a significant difference between groups (*P* < 0.05). As shown in Fig. 6B,C and Supplementary files Table S4, a total of 24 species, which represented 11 genera, were significantly different between groups (*P* < 0.05). At the genus level, they are *g-Anaeroplasma*, *g-Anaerovorax*, *g-Bacteroides*, *g-Desulfovibrio*, *g-Dorea*, *g-Enterorhabdus*, *g-Helicobacter*, *g-Odoribacter*, *g-Parabacteroides*, *g-Paraprevotella* and *g-Prevotella*. Among them, an increased relative abundance of *g-Enterorhabdus* was detected in the model group compared with that in the normal group (*P* < 0.05). Betaine downregulated the relative abundance of *g-Enterorhabdus* (*P* < 0.05). The relative abundance of *g-Bacteroides* was the highest in the normal group and the lowest in the model group (*P* < 0.05). The relative abundance of *g-Prevotella* was almost the same in the normal and betaine groups and was reduced in the betaine group (*P* < 0.05).

### Species classification and abundance analysis

**Figure 7:**
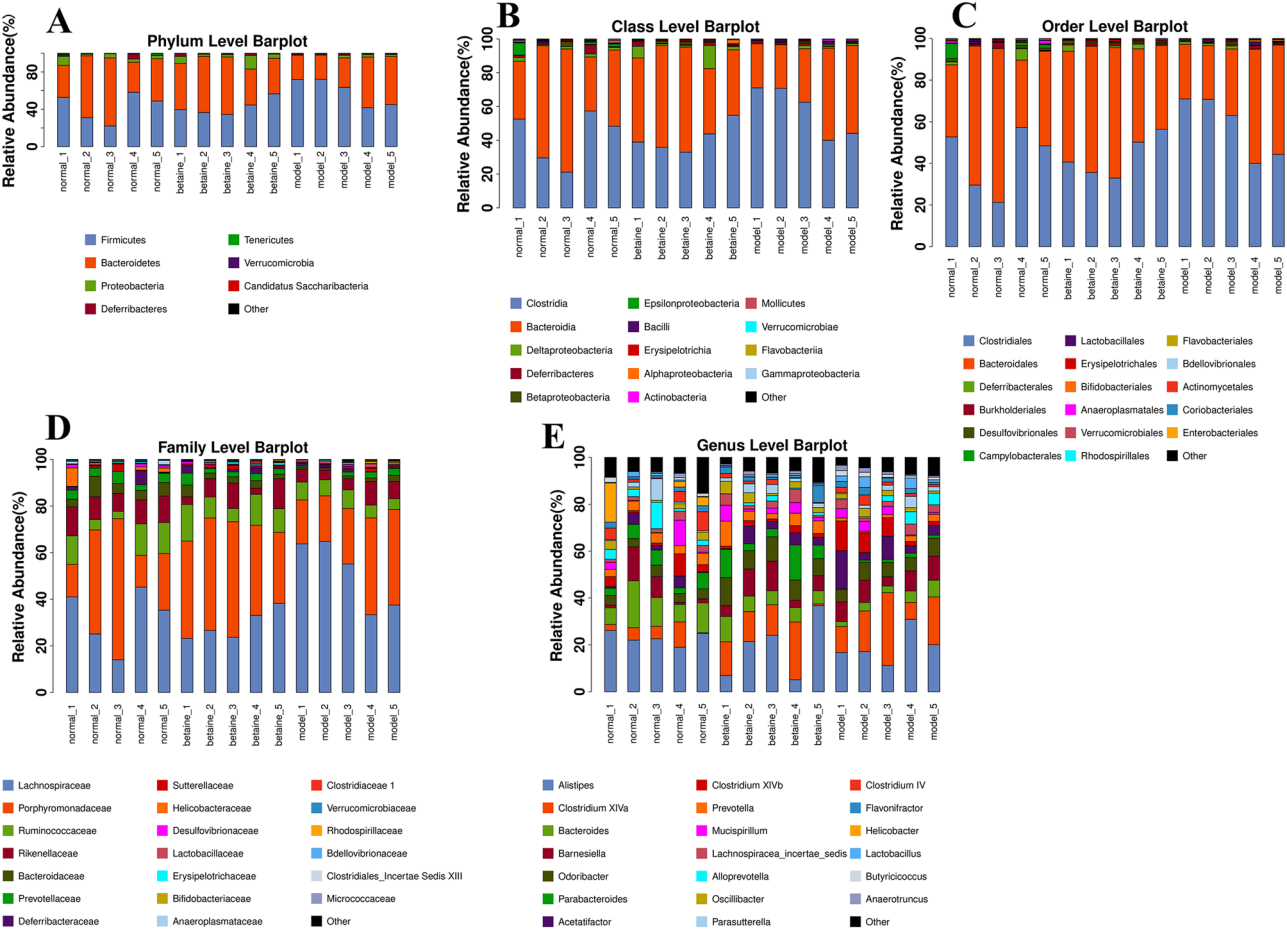
Species classification and abundance analysis. (**A**–**E**) The corresponding histograms of species profiling were produced for each sample at the classification level of phylum, class, order, family, and genus.

**Figure 8:**
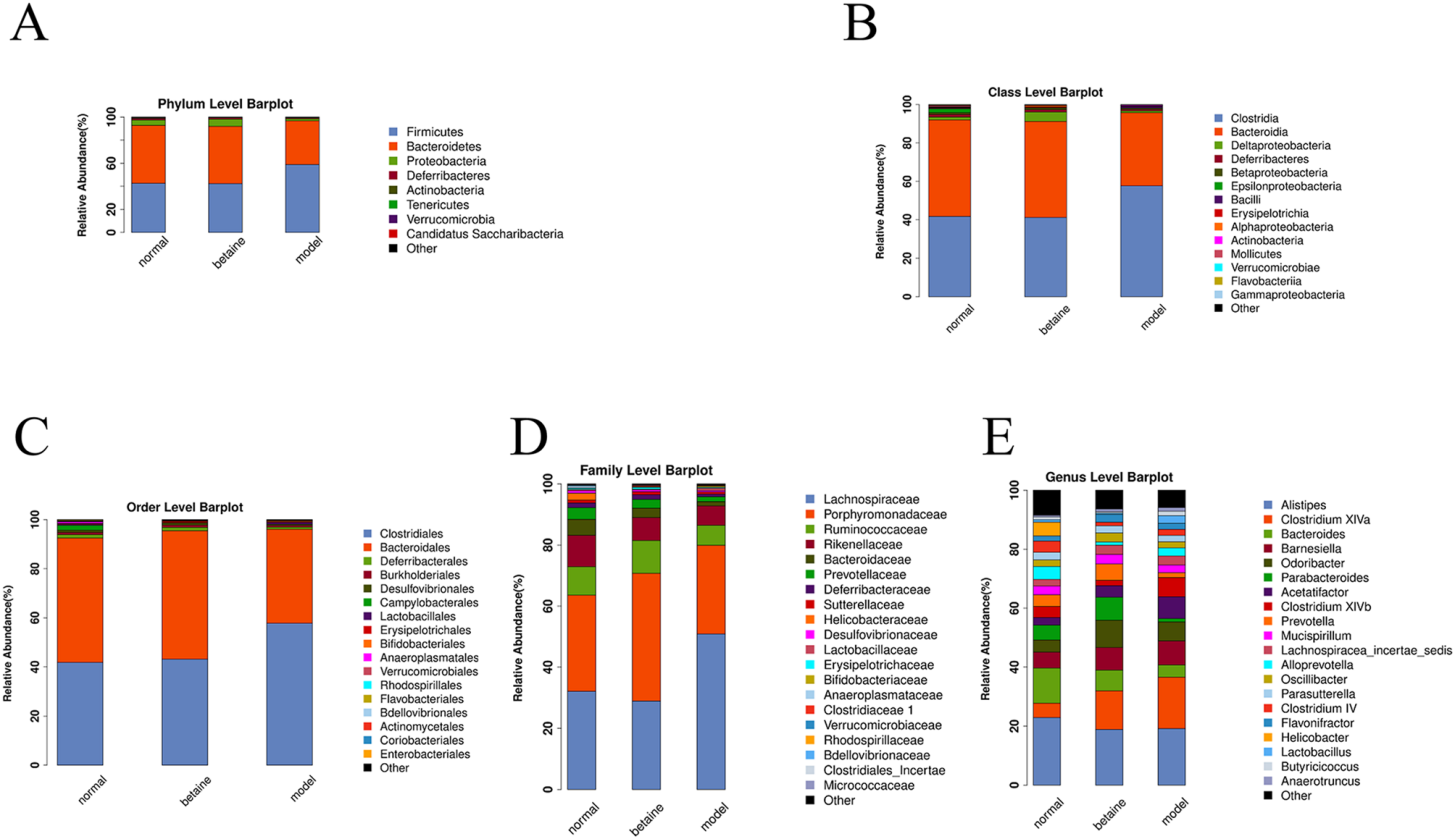
Species classification and abundance analysis. (**A**–**E**) The corresponding histograms of species profiling were produced for each group at the classification level of phylum, class, order, family, and genus.

A sequence with the highest abundance was selected from each OTU as a representative sequence of the OTU. Using the RDP method, the representative sequence was aligned with the 16S database to classify each OTU to a corresponding species. Forming a relative abundance histogram of species, we visually observed the proportion of different species abundances in each sample and group. As shown in Figs. 7A–E, 8A–E, at the classification level of phylum, class, order, family, and genus, the corresponding histograms of microbiome species profiling were produced for each sample and group. The majority of the microbiome at the phylum level among each group belonged to *Firmicutes*, *Bacteroidetes*, *Proteobacteria*, *Deferribacteres*, *Actinobacteria*, *Tenericutes*, *Verrucomicrobia* and *Candidatus Saccharibacteria*. Surprisingly, the majority of the microbiome phyla occupied similar proportions between the normal group and the model group. In addition, *Firmicutes* was the most abundant phylum in each group. The relative abundance of *Firmicutes* in model mouse feces (58.8%) was significantly higher than that in the normal (42.5%) and betaine group mouse feces (42.3%) (*P* < 0.05). A previous study confirmed that the levels of *Firmicutes* were positively correlated with ACLF severity and that the abundance of *Bacteroidetes* was inversely correlated. It has been suggested that betaine plays a role in potentially modifying the gut bacterial community. The relative abundance of *Bacteroidetes* in model mouse feces (37.8%) was apparently lower than that in the normal (50.2%) and betaine group mouse feces (49.5%) (*P* < 0.05) (Fig. 8A and Supplementary files Table S5). The relative abundance of *Alistipes* (belonging to *Bacteroidetes*) was enriched in the normal group (22.9%) and decreased in the betaine (18.8%) and model groups (19.1%) (*P* < 0.05). Moreover, compared with that in the normal group, the relative abundance of *Clostridium XlVa* (belonging to *Bacteroidetes*) was significantly higher in the model group. Betaine pretreatment reduced the relative abundance of *Clostridium XlVa* in the model group (*P* < 0.05) (Fig. 8E and Supplementary files Table S6). In general, the abundances of the gut microbiota taxa *Coriobacteriaceae*, *Lachnospiraceae*, *Enterorhabdus* and *Coriobacteriales* were remarkably increased in the model group, contrary to those of the gut microbiota taxa *Bacteroidaceae*, *Bacteroides*, *Parabacteroides* and *Prevotella*. Betaine significantly altered the microbial communities, depleted the gut microbiota constituents *Coriobacteriaceae, Lachnospiraceae, Enterorhabdus* and *Coriobacteriales* and markedly enriched the taxa *Bacteroidaceae, Bacteroides, Parabacteroides* and *Prevotella* (*P* < 0.05) (Fig. 8A–E)*.* These results indicated that alteration of the gut microbiome might be a crucial therapeutic target in ALF.

## Discussion

Since 1998, when Marshall proposed the gut-liver axis, much attention has been paid to the role of the gut in liver disease^30^. Systemic endotoxemia, characterized by increased plasma LPS concentrations, increased intestinal permeability by altering tight junctions and thus resulted in more endotoxins entering the portal vein and activating Kupffer cells in the liver. This effect results in the production of proinflammatory cytokines and acute inflammatory response proteins, which persistently aggravate hepatic insufficiency and/or failure^2^. In addition, our previous study indicated that intestinal injury plays a vital role in ALF progression^27^. All these results support that if intestinal injury was reduced, liver lesions would be alleviated in ALF. The gut-liver axis plays an essential role in the pathogenesis of ALF.

Epithelial cells are held together by the apical junctional complex, which includes transmembrane proteins (claudins and occludin) and the cytosolic scaffold proteins (ZO)-(1–3) and cingulin. Research has proven that inflammatory conditions contribute to significant disturbance of mucosal barrier function, with decreased expression and redistribution of claudin, (ZO)-1, and occludin, which lead to increased permeability^31^. LPS, which can trigger a powerful inflammatory response, is recognized as a causal or complicating factor of multiple serious diseases. One of the mechanisms that prevents LPS from transiting from the intestine into the systemic circulation is through TLRs, which can activate the immune system^32^. However, the continuous stimulation of TLR signaling does not always exert positive impacts on the host, and it can enhance hepatic injury in chronic viral hepatitis, NASH, and alcoholic liver disease^33–35^. In addition, the reorganization of (ZO)-1 in tight junctions is activated by TLRs. There are 11 TLRs that have been identified in mammals, whereas LPS binds mainly TLR4. The MyD88-dependent pathway, as one of the downstream signaling events mediated by TLR4, leads to the recruitment of numerous molecules that activate tumor necrosis factor (TNF-α) and other proinflammatory factors^8^. TNF-α can downregulate the transmembrane tight junction protein expression of occludin, paralleling the barrier disturbance detected electrophysiologically, which leads to an increase in intestinal permeability^36^. In our animal and cell experiments, the results indicated that mRNA and protein level of TLR4 and MyD88 were increased in intestine induced by LPS. And the levels of TNF-α, IL-1β and IL-18 in serum, liver and small intestine were greatly increased in the ALF model group. However, the protein and mRNA level of (ZO)-1 and occludin were decreased in intestine stimulated by LPS. The intestinal permeability is increased in ALF model. Our data also proved that TLR4/MyD88 signaling pathway played a vital role in intestine injury in ALF which was consistent with previous findings^37^.

The intestinal epithelial barrier is not a static physical barrier but rather strongly interacts with immune system cells and the gut microbiome. Under physiological conditions, there is a dynamic regulation of tight junction components in the intestine. However, sustaining infections or inflammation can result in dysregulation in the expression of adhesion molecules, bringing about barrier breach and disturbance of gut microbes^38^. Studies have demonstrated that TLRs are innate pattern recognition receptors involved in host defense, preference for normal over commensal bacteria and the maintenance of tissue integrity^39^. An experiment was performed with MyD88-negative mice with a certain microbial consortium representing bacterial phyla normally existing in the human gut^40^. It was found that *B*. *adolescentis* exhibited antiinflammatory properties in D-Gal-treated rats. The gut microbiota is involved in this process. A study showed that *S. boulardii* notably reduced the relative abundance of the phylum *Bacteroidetes* by enhancing the relative abundances of *Firmicutes* and *Proteobacteria* in ALF mice^41^.

Betaine, an oxidative metabolite of choline, can protect rats from induction of LPS hepatotoxicity^42^. Betaine can be used as a methyl donor, catalyzed by betaine-homocysteine methyltransferase, to re-methylate homocysteine to produce methionine, thereby reducing homocysteine level. At present, betaine is not used clinically. But betaine widely exists in nature, such as in spinach. Moreover, significant amount of data from animal models of liver disease indicates that administration of betaine can halt and even reverse progression of the disruption of liver function^43^. The present study showed that betaine exerts positive effects on the gut-liver axis, including inhibition of potent inflammatory responses and maintenance of gut integrity. Moreover, in cell experiments, the dose of betaine was positively correlated with its protective effect in the model group. In our study, 16S rRNA gene sequence analysis was used to explore the potential therapeutic microecological mechanisms of betaine in ALF mice. There were 156 core microbiome constituents in fecal samples. The abundances of the gut microbiota taxa *Coriobacteriaceae*, *Lachnospiraceae*, *Enterorhabdus* and *Coriobacteriales* were remarkably increased in the model group, contrary to those of the gut microbiota taxa *Bacteroidaceae*, *Bacteroides*, *Parabacteroides* and *Prevotella*. Betaine significantly increased the microbial communities, depleted the gut microbiota constituents *Coriobacteriaceae, Lachnospiraceae, Enterorhabdus* and *Coriobacteriales* and markedly enriched the taxa *Bacteroidaceae, Bacteroides, Parabacteroides* and *Prevotella.* What’s more, the previous study has also demonstrated that *Bacteroides* and *Prevotella* were depleted in D-Gal-induced liver injury^44^. Based on available research, *Prevotella* is beneficial to glucose metabolism and improve hepatic glycogen storage^45^*. Prevotella* is suggested to exert protective effects against the development of NAFLD and to be more abundant in healthy subjects than that in NAFLD patients^46^. *Bacteroides* can ferment undigested polysaccharides and is the most predominant anaerobe in the gut^47^. The inflammatory cytokines level of TNF-α was negatively correlated with *Bacteroidetes* in ACLF patients^17^. And our results showed that *Bacteroidetes* was significantly decreased and the level of TNF-α was markedly increased in ALF mouse. Besides, *Enterorhabdus*, a member of the family *Coriobacteriaceae*, is isolated from a mouse model of spontaneous colitis^48^. Several reports found that the *Lachnospiraceae* is enhanced in NALFD patients and it contributes to the development of obesity and diabetes in ob/ob mice^49^. In short, administration of betaine modifies the gut bacterial community, enriching beneficial microbial taxa and inhibiting opportunistic pathogens, and this effect may help ameliorate liver failure.

## Conclusion

Betaine improved liver function, liver and small intestine histology, and intestinal permeability and consolidated the tight junction between small intestine epithelial cells. The present study not only proved that betaine had hepatoprotective effects in ALF mice but also further demonstrated its protective effects on the structure and function of the small intestine. One of the protective effects of betaine on the small intestine in ALF occurs via inhibition of the LPS/TLR4/MyD88 pathway, improving intestinal permeability and ultimately dramatically shaping the gut microbiota in mice. This work revealed a new role for betaine in improving hepatic lesions. These findings thus suggested that the compounds targeted in the gut-liver axis should be further investigated as novel adjunctive therapies for ALF. Additional clinical studies are needed to verify an effective strategy to prevent and manage ALF by altering the gut bacterial community.

## Supplementary Information

Supplementary Tables.
