# Supplementary material for: Betaine inhibits Toll-like receptor 4 responses and restores intestinal microbiota in acute liver failure mice
Source: Sci Rep. 2020 Dec 14;10:21850. doi: 10.1038/s41598-020-78935-6 (PMC7736280; doi:10.1038/s41598-020-78935-6)
Supplement: Supplementary file 1 — Supplementary Tables. [file 41598_2020_78935_MOESM1_ESM.pdf]

Betaine Inhibits Toll-like Receptor 4 Responses and Restores Intestinal Microbiota in Acute Liver Failure Mice

Qian Chen, Yao Wang, Fangzhou Jiao, Chunxia Shi, Maohua Pei, Luwen Wang, and Zuojiong Gong,
Department of Infectious Diseases, Renmin Hospital of Wuhan University, 430060 Wuhan, China
Zuojiong Gong
E-mail: zjgong@163.com
Telephone: +86 027-88041911-88385
Fax: +86 27-88042292

Table S1: OTU network analyses showed 509 specific OTUs were produced from 15 fecal samples.

#OTU ID	normal_4	betaine_5	normal_1	model_4	normal_5	model_1	model_5	normal_3	betaine_4	model_3	model_2	normal_2	betaine_3	betaine_1	betaine_2
OTU434	1	4	3	1	1	4	1	0	0	0	0	0	0	0	0
OTU501	0	0	0	1	0	7	3	2	4	1	0	0	0	0	0
OTU500	1	1	8	4	27	3	0	1	0	3	1	4	0	0	0
OTU503	0	1	0	3	1	1	0	1	0	0	2	0	1	1	0
OTU502	0	0	1	0	2	0	0	0	1	0	0	0	0	1	2
OTU505	6	22	76	12	52	11	14	1	9	54	5	7	0	28	4
OTU504	0	0	3	0	1	0	0	0	0	0	0	0	0	1	0
OTU507	53	12	89	18	3	6	7	1	7	1	309	7	12	4	18
OTU506	0	0	0	0	0	0	0	1	0	0	0	0	0	1	6
OTU509	0	1	3	1	1	0	0	1	0	3	0	0	0	0	1
OTU508	0	0	0	0	0	0	0	2	0	0	0	0	0	0	0
OTU480	3	0	1	0	0	0	0	0	0	0	0	0	0	0	0
OTU481	6	0	1	0	0	0	0	0	0	0	0	1	0	0	0
OTU482	0	7	0	0	0	0	0	0	1	0	0	0	1	0	0
OTU483	0	0	4	2	1	5	2	1	0	3	3	2	1	0	0
OTU484	0	1	0	0	0	0	0	0	1	0	0	0	0	1	0
OTU485	1	1	3	3	2	1	1	0	0	5	4	0	3	0	1
OTU338	7	3	11	15	3	4	5	4	5	6	6	14	9	8	4
OTU339	0	0	0	6	0	4	9	0	0	31	0	4	0	2	2
OTU336	3	1	17	6	15	0	4	1	5	6	7	4	3	3	3
OTU337	12	43	13	13	12	3	26	15	45	15	8	9	34	20	25
OTU334	9	0	30	0	30	0	0	1	2	0	0	17	0	0	0
OTU335	1	0	17	0	12	0	0	0	0	0	0	2	0	0	0
OTU332	5	12	39	14	4	18	5	7	7	4	12	6	11	3	0
OTU333	14	1	12	3	5	10	7	2	6	9	8	0	4	9	7
OTU330	6	6	0	1	3	7	2	0	0	2	1	1	3	14	10
OTU331	5	8	0	8	0	7	8	3	0	5	6	0	7	1	6
OTU349	4	2	9	6	14	14	5	2	3	3	9	5	1	2	1

OTU348	2	2	9	3	8	1	6	2	0	2	2	1	9	2	6
OTU439	3	0	7	0	11	0	0	0	0	0	0	4	0	0	0
OTU438	0	2	0	4	1	0	1	1	0	1	0	3	4	0	1
OTU343	0	1	0	4	0	6	8	2	2	2	0	1	4	1	4
OTU342	10	11	21	11	28	9	16	10	13	16	4	18	28	14	14
OTU341	1	0	16	0	19	0	0	0	0	0	0	11	0	0	0
OTU340	0	0	0	0	0	0	1	2	0	0	0	0	1	1	34
OTU347	2	0	0	0	1	15	0	0	2	1	3	3	1	0	2
OTU346	0	14	0	1	0	0	2	6	0	0	1	4	2	8	3
OTU345	6	7	8	2	9	10	1	6	2	4	21	1	1	1	3
OTU344	5	2	7	2	2	2	4	1	3	17	7	0	7	6	1
OTU158	53	1	108	23	65	92	23	23	1	11	332	23	0	1	2
OTU159	22	21	171	20	89	92	14	11	9	37	140	22	2	8	12
OTU152	3	0	0	22	0	3	130	13	0	246	1	0	0	0	0
OTU153	26	1	134	16	17	1	30	3	3	71	136	5	2	0	5
OTU150	86	338	59	86	129	21	86	133	165	59	2	58	76	45	42
OTU151	18	1	10	31	3	18	2	116	0	1	159	4	3	2	7
OTU156	8	134	41	9	55	6	0	0	0	1	25	9	0	2	2
OTU157	74	0	8	0	100	0	0	0	0	0	0	51	0	0	0
OTU154	32	104	0	15	0	27	30	35	49	60	30	5	59	177	43
OTU155	22	4	90	3	121	9	7	34	0	9	7	34	2	0	1
OTU237	3	37	0	1	0	0	5	5	0	12	0	5	6	0	1
OTU236	38	8	19	16	21	19	4	5	0	9	25	6	18	0	10
OTU235	3	3	5	2	7	1	2	3	3	4	0	2	2	33	4
OTU234	11	2	21	25	0	129	13	8	19	30	11	8	9	13	7
OTU233	2	39	0	10	3	3	31	52	46	31	7	14	51	4	21
OTU232	0	16	0	6	0	2	11	5	1	3	2	18	44	44	72
OTU231	0	42	18	8	6	1	5	4	7	6	7	3	17	1	6
OTU230	84	0	22	0	11	2	0	3	0	0	3	4	0	0	0
OTU239	28	6	48	14	54	8	2	2	10	8	22	6	1	10	3
OTU238	162	36	103	52	142	134	16	53	20	56	135	27	29	94	51
OTU61	86	137	6	440	42	37	179	845	169	264	95	174	423	184	386
OTU60	5	18	0	23	1	1	537	48	927	719	1	8	313	7	358
OTU63	0	104	0	6	0	0	3	23	508	6	4	3	13	2	0
OTU62	20	821	10	22	9	2	247	7	0	35	11	32	387	0	26
OTU65	666	418	150	162	345	172	37	42	193	150	172	96	127	230	104
OTU64	218	0	435	0	413	1	0	0	0	0	2	122	0	1	1
OTU67	44	60	67	58	60	41	21	34	155	56	58	38	26	435	177
OTU66	64	162	20	97	59	72	250	345	141	142	68	89	216	50	180
OTU69	5	23	0	0	2	0	1	6	512	0	0	0	8	124	4
OTU68	49	553	120	31	17	46	31	20	60	15	38	21	70	165	33

OTU471	0	6	0	1	1	0	0	0	0	0	0	0	0	0	0
OTU470	0	2	0	1	0	0	0	0	1	0	0	1	0	3	4
OTU473	3	0	0	0	0	2	1	0	3	0	1	2	13	1	5
OTU472	0	0	0	3	0	1	0	0	0	1	6	6	0	0	0
OTU475	0	0	0	0	0	0	0	3	0	0	0	0	0	0	0
OTU474	1	0	0	0	0	0	0	0	1	0	0	0	0	1	0
OTU477	0	0	1	1	0	36	0	0	0	0	1	1	0	0	0
OTU476	1	0	3	2	3	2	2	0	0	1	2	1	0	0	0
OTU479	97	4	3	4	13	50	12	6	16	32	23	2	10	0	25
OTU478	0	4	0	2	0	0	0	0	3	2	0	0	0	0	2
OTU208	9	4	3	21	3	80	19	6	16	31	88	4	2	4	3
OTU209	39	8	27	9	55	26	11	11	9	30	17	18	0	3	4
OTU206	88	16	180	18	108	14	36	17	22	24	29	31	29	37	42
OTU207	12	12	2	56	2	18	12	31	2	10	10	25	11	0	7
OTU204	10	55	15	3	5	11	18	6	26	33	11	5	17	77	8
OTU205	38	8	23	22	16	10	30	12	1	40	3	2	2	11	0
OTU202	15	0	71	0	65	0	0	0	0	0	0	25	0	0	0
OTU203	11	0	31	0	66	0	0	0	0	0	1	32	0	0	0
OTU200	0	24	0	42	0	0	2	2	15	3	2	19	18	19	10
OTU201	0	68	0	1	4	16	0	5	0	0	0	0	0	0	0
OTU486	8	41	2	9	0	0	6	3	0	11	0	5	5	5	6
OTU487	0	0	0	0	0	0	0	0	2	0	0	0	0	1	0
OTU372	0	4	0	12	1	5	4	4	1	1	5	6	3	0	4
OTU373	0	1	0	1	0	0	2	9	0	4	3	7	0	0	0
OTU279	28	0	24	0	19	0	0	1	1	0	0	6	0	0	0
OTU278	0	0	26	0	0	2	1	0	1	0	0	0	0	0	1
OTU376	3	0	0	0	0	0	3	11	0	10	1	0	0	0	1
OTU377	2	0	0	3	0	0	0	0	0	0	0	0	0	2	0
OTU374	4	1	1	1	1	3	3	2	0	4	0	11	15	0	2
OTU375	2	9	1	6	6	3	4	0	2	6	2	8	4	2	4
OTU273	10	2	12	2	15	18	6	3	9	14	3	8	12	33	11
OTU272	53	43	22	24	33	38	47	48	155	29	27	18	41	241	86
OTU271	5	4	0	15	1	1	2	39	1	8	1	13	3	0	0
OTU270	2	2	0	10	0	0	9	0	0	1	34	0	9	0	30
OTU277	0	0	0	7	0	60	2	1	2	14	6	4	1	0	5
OTU276	39	0	85	8	43	27	6	1	0	11	19	17	0	0	0
OTU275	9	11	0	8	0	2	5	0	4	17	33	25	1	13	3
OTU274	8	0	5	0	26	0	0	0	0	0	0	19	0	0	0
OTU105	183	88	304	64	269	117	55	40	143	155	133	54	48	170	189
OTU104	16	88	9	126	34	42	74	228	208	17	27	211	331	87	298
OTU107	0	109	0	2	0	0	25	22	167	32	0	2	8	40	22

OTU106	17	62	7	237	28	105	208	148	53	63	206	200	264	36	202
OTU101	42	1	410	44	31	107	9	1	0	66	13	16	2	2	3
OTU100	48	217	49	13	55	0	15	11	4	21	4	61	6	50	40
OTU103	15	0	174	31	311	150	16	2	0	37	1	61	0	0	1
OTU102	12	0	0	46	2	373	31	31	2	56	0	152	0	4	0
OTU109	115	0	243	0	153	0	0	0	0	0	0	29	0	0	0
OTU108	93	12	34	37	65	122	91	12	35	256	22	26	39	113	115
OTU295	3	0	0	2	0	35	8	3	1	17	0	3	0	0	0
OTU294	2	15	1	8	0	1	7	11	14	3	2	2	13	8	10
OTU297	0	0	3	1	8	3	3	0	0	2	4	6	0	1	1
OTU296	20	0	30	1	4	4	0	0	3	4	3	2	2	0	1
OTU291	11	7	16	9	11	15	4	3	5	9	23	1	4	8	1
OTU290	33	21	22	17	14	17	28	2	6	34	14	9	5	4	9
OTU293	14	2	4	1	9	0	2	4	2	2	2	15	3	3	2
OTU292	0	1	0	6	0	0	3	53	0	2	1	10	2	0	0
OTU299	11	2	7	4	12	3	1	4	11	11	15	5	7	9	6
OTU298	2	12	22	8	15	6	15	4	5	8	9	3	4	19	1
OTU198	33	10	28	9	2	11	46	10	3	20	41	18	7	8	3
OTU199	0	2	0	0	0	0	0	0	1	0	0	0	1	53	0
OTU196	458	0	102	2	85	0	0	0	0	0	0	401	0	2	0
OTU197	1	76	0	12	1	0	23	2	26	3	4	3	44	9	9
OTU194	1	0	3	19	4	4	25	6	0	68	2	0	0	0	0
OTU195	12	12	34	1	57	16	0	0	0	3	0	4	9	0	8
OTU192	14	8	24	19	57	14	12	8	12	15	5	24	24	69	22
OTU193	89	0	29	0	26	0	0	0	0	0	0	5	0	0	0
OTU190	11	0	84	0	6	0	0	0	0	0	0	0	0	0	0
OTU191	16	7	36	40	17	37	54	7	0	22	35	41	92	1	57
OTU38	2	7	55	952	6	143	1108	498	1	1167	3	1568	0	0	5
OTU39	281	347	317	85	767	20	84	389	1660	96	47	608	229	1120	192
OTU36	204	177	177	636	236	135	564	785	21	306	34	470	199	147	115
OTU37	128	5	873	1	381	21	4	2	0	13	10	22	0	20	3
OTU34	47	223	8	74	4	8	149	275	1507	186	2	12	46	1248	11
OTU35	1344	102	528	108	356	1209	122	75	304	845	533	101	77	63	126
OTU32	47	377	0	374	45	12	443	509	353	182	32	123	512	539	740
OTU33	28	9	113	6	64	9	153	15	17	104	3	61	826	1	29
OTU30	456	5	952	523	587	86	40	99	21	13	73	508	77	452	40
OTU31	165	100	536	493	276	46	491	1060	55	296	40	383	266	5	131
OTU371	11	1	7	2	26	2	2	0	0	0	0	4	2	1	2
OTU435	23	2	0	8	2	67	67	5	0	229	9	7	4	0	2
OTU378	6	2	0	0	0	0	0	1	0	11	2	0	2	0	1
OTU379	0	0	0	0	0	10	1	11	0	0	0	0	0	0	0

OTU83	33	175	5	66	14	11	59	83	300	79	6	56	139	131	50
OTU82	79	117	44	69	66	73	54	125	386	67	76	77	106	631	128
OTU81	2	1	376	21	254	35	33	80	56	14	261	151	23	26	66
OTU80	45	39	14	404	35	225	61	87	11	27	283	246	58	52	43
OTU87	5	2	1	221	15	11	71	70	6	52	765	2	54	5	27
OTU86	253	557	0	55	8	40	72	12	74	95	55	18	116	37	162
OTU85	35	37	0	256	16	3	186	80	37	76	38	52	77	39	91
OTU84	250	108	297	6	317	28	4	24	22	27	121	39	6	28	20
OTU89	28	105	20	38	33	21	102	379	45	52	14	26	51	67	38
OTU88	8	133	0	12	7	0	37	19	193	45	1	5	228	198	451
OTU329	10	0	14	0	1	0	0	0	0	0	0	1	0	0	0
OTU328	3	6	5	5	8	3	7	4	11	4	1	5	6	6	9
OTU499	0	3	1	1	3	1	2	0	0	1	1	2	0	0	0
OTU498	0	1	0	4	0	1	1	0	0	0	0	0	1	0	1
OTU325	17	2	0	3	0	6	4	2	0	3	1	1	21	0	10
OTU324	4	0	103	0	214	0	1	0	8	0	0	18	0	2	0
OTU327	1	0	1	10	0	0	1	1	0	3	1	1	1	2	1
OTU326	1	0	0	12	1	2	5	11	1	14	1	20	7	0	3
OTU321	0	0	16	0	2	0	0	0	0	0	0	2	0	0	0
OTU320	0	5	0	3	0	7	0	0	1	0	1	27	8	0	1
OTU323	15	3	10	9	0	19	10	3	9	8	6	9	6	16	1
OTU322	0	0	0	0	0	0	0	0	0	0	0	0	0	20	1
OTU408	2	0	2	1	1	2	0	0	0	2	4	0	0	1	0
OTU409	0	0	1	0	0	3	3	0	0	1	1	1	0	1	0
OTU400	3	2	1	0	0	5	1	1	0	0	2	1	3	1	4
OTU401	5	0	0	2	0	0	11	3	0	19	2	3	0	0	1
OTU402	3	0	2	3	2	2	1	0	4	1	0	3	1	1	2
OTU403	5	2	12	2	5	8	3	0	4	5	7	1	0	3	2
OTU404	0	1	0	0	0	0	5	0	2	3	1	0	1	4	0
OTU405	1	1	2	6	3	5	4	0	1	3	0	4	2	1	2
OTU406	0	0	0	0	0	0	0	0	0	1	6	6	0	0	0
OTU407	161	1	3	43	10	211	27	135	90	27	39	80	77	473	70
OTU149	9	0	13	1	12	0	0	0	127	0	0	7	1	30	100
OTU148	94	71	23	70	24	159	27	35	34	26	61	35	26	19	32
OTU141	69	0	0	3	2	158	6	14	0	22	13	19	0	0	1
OTU140	107	63	110	60	42	1	61	8	93	42	1	48	44	31	29
OTU143	29	78	14	13	23	12	108	28	15	29	3	21	42	45	32
OTU142	23	11	0	2	4	3	6	9	134	7	6	2	4	1	2
OTU145	5	15	0	71	2	10	31	1	0	120	442	11	0	1	0
OTU144	16	10	5	18	28	6	31	24	18	6	3	34	47	98	82
OTU147	2	3	105	3	126	58	4	2	0	17	4	19	5	0	1

OTU146	5	65	1	1	4	3	122	1	152	88	4	5	34	57	11
OTU390	2	0	14	2	8	0	0	1	3	4	3	14	0	0	0
OTU391	5	0	0	0	0	0	4	0	0	12	1	2	0	6	3
OTU392	2	2	5	6	4	10	1	2	0	2	3	4	0	3	1
OTU393	8	0	8	0	3	0	0	0	0	0	0	2	0	0	0
OTU394	3	1	0	1	0	0	1	0	5	0	0	1	2	1	0
OTU395	1	4	0	1	2	0	0	1	2	0	0	3	5	4	2
OTU396	3	0	5	0	1	0	0	0	0	0	0	1	0	0	0
OTU397	7	0	9	10	6	12	2	1	0	3	6	7	0	1	0
OTU398	10	6	69	2	1	308	2	1	116	1	7	1	3	1	0
OTU399	1	1	6	0	12	0	3	0	0	0	0	6	1	1	0
OTU437	39	7	17	7	8	9	13	18	14	15	2	6	12	2	45
OTU436	9	3	229	47	94	46	57	4	42	6	98	8	29	3	16
OTU431	0	0	4	0	12	0	0	0	0	0	0	0	0	0	0
OTU430	0	1	0	3	0	0	0	1	2	1	0	2	2	2	4
OTU242	19	13	43	9	10	19	5	1	17	4	11	7	15	28	32
OTU243	0	0	0	1	0	0	0	0	38	0	0	0	4	2	1
OTU240	10	0	43	0	37	0	0	0	0	0	0	1	0	0	0
OTU241	19	0	37	0	31	0	0	0	0	0	0	59	0	0	0
OTU246	22	6	22	11	8	23	11	1	23	23	17	14	22	18	39
OTU247	15	4	11	55	6	5	10	8	34	14	110	2	1	14	2
OTU244	1	3	0	5	0	56	0	0	4	5	0	0	1	0	1
OTU245	3	8	102	21	12	61	6	1	0	13	93	10	8	0	5
OTU248	33	0	5	19	7	0	46	0	0	40	1	3	2	0	0
OTU249	34	0	41	0	33	0	0	1	1	0	0	26	0	0	0
OTU433	76	0	119	0	265	1	5	0	1	0	0	12	1	2	5
OTU72	31	58	9	52	19	9	26	156	37	37	15	43	76	345	65
OTU73	16	8	245	18	508	2	25	20	11	8	4	40	16	15	11
OTU70	234	107	55	450	130	556	73	54	54	162	445	123	126	86	120
OTU71	141	15	461	55	221	134	75	17	48	124	106	27	36	43	140
OTU76	120	26	0	29	4	200	108	25	0	53	3	73	138	12	318
OTU77	214	125	110	91	59	65	50	39	139	116	75	58	81	260	116
OTU74	115	59	55	99	61	99	74	35	151	236	83	28	72	288	216
OTU75	22	12	414	57	26	98	8	20	3	15	482	35	3	3	5
OTU78	59	1	0	382	7	269	96	4	0	40	744	35	0	0	0
OTU79	78	21	0	97	1	19	205	35	106	105	32	60	63	87	65
OTU432	2	4	0	0	0	1	5	0	6	1	2	1	1	0	3
OTU448	82	311	2	132	7	283	57	111	5	5	30	28	46	15	57
OTU449	0	0	0	4	1	38	2	2	0	3	0	19	4	4	5
OTU444	1	3	0	2	0	4	1	0	0	3	5	3	0	0	1
OTU445	0	0	0	0	0	1	0	0	0	0	3	0	0	0	0

OTU446	0	0	0	2	1	4	2	1	0	1	1	0	0	0	0
OTU447	0	0	0	0	0	0	0	0	0	0	0	1	0	0	0
OTU440	73	0	0	13	0	4	81	2	0	55	13	1	3	1	4
OTU441	0	6	0	0	0	1	0	0	0	0	0	0	0	0	0
OTU442	5	1	4	2	4	3	2	0	2	0	5	3	3	1	1
OTU443	4	4	5	1	0	5	0	1	8	0	3	3	2	0	0
OTU219	63	1	43	8	163	34	10	9	3	13	5	12	0	2	0
OTU218	5	0	52	0	54	0	0	0	0	0	0	42	0	0	1
OTU215	43	57	2	36	17	6	21	63	99	12	6	34	76	38	48
OTU214	4	0	32	0	71	0	0	0	0	0	0	67	0	0	0
OTU217	24	127	6	53	19	31	76	138	118	44	17	82	203	83	68
OTU216	11	37	10	21	8	17	23	14	45	50	8	22	58	9	17
OTU211	4	9	9	0	39	0	1	0	0	5	0	42	0	0	0
OTU210	111	36	133	19	51	167	16	10	4	33	45	29	1	6	6
OTU213	30	33	106	55	104	82	23	24	24	43	135	30	21	16	23
OTU212	1	20	40	3	30	0	12	21	14	2	0	36	8	0	1
OTU496	1	0	6	0	7	0	0	0	0	0	0	3	0	0	0
OTU361	3	1	6	3	2	25	4	1	0	12	26	4	0	0	0
OTU360	0	4	1	3	3	4	0	3	0	0	0	1	9	22	1
OTU363	1	0	0	1	0	26	1	0	2	15	0	0	1	1	3
OTU362	0	2	0	2	0	2	1	2	2	3	0	10	4	2	4
OTU365	3	0	2	1	1	42	0	0	7	0	4	1	0	1	0
OTU364	313	453	57	230	15	19	116	174	9	321	31	135	84	109	121
OTU367	5	14	0	5	0	0	1	1	0	2	3	1	0	1	2
OTU366	3	0	8	0	3	0	0	0	0	0	0	0	0	0	0
OTU369	1	0	1	0	19	0	0	0	0	0	0	3	0	0	0
OTU368	9	2	2	5	7	7	1	0	0	5	0	9	1	2	0
OTU303	2	0	9	3	0	0	3	0	0	1	7	9	5	4	20
OTU130	142	105	22	63	5	81	81	142	9	119	50	15	100	63	33
OTU131	28	32	12	166	52	97	142	222	41	120	46	99	138	13	150
OTU132	72	133	1	1	17	4	2	2	8	6	7	33	5	19	32
OTU133	5	211	134	28	100	1	24	31	62	42	58	54	97	8	27
OTU134	8	3	0	1	0	0	0	0	177	1	5	0	5	313	2
OTU135	87	29	58	29	117	70	34	11	23	40	48	38	38	22	80
OTU136	10	68	93	157	38	35	64	125	71	30	35	168	169	376	188
OTU137	0	0	181	0	10	0	0	0	0	0	0	19	0	0	0
OTU138	61	87	1	9	15	6	42	95	77	157	44	4	170	39	132
OTU139	71	6	47	51	127	1	4	19	55	0	3	147	2	1	0
OTU189	1	0	0	7	0	75	3	1	1	2	25	13	0	0	0
OTU188	32	82	4	4	8	0	21	5	0	38	0	1	44	4	1
OTU185	11	8	0	3	0	296	9	2	16	62	0	4	1	0	2

OTU184	49	19	118	19	111	43	8	5	4	28	51	19	1	1	4
OTU187	95	0	125	0	131	0	0	1	0	0	0	165	0	0	0
OTU186	12	0	75	0	63	0	0	0	0	0	0	52	0	0	0
OTU181	79	16	15	0	53	0	0	0	3	1	0	10	0	1	0
OTU180	20	69	9	39	196	3	2	125	49	16	1	13	31	103	2
OTU183	48	32	22	13	25	26	10	6	76	76	12	6	12	45	23
OTU182	34	7	162	37	217	44	21	11	13	29	18	53	18	16	21
OTU493	2	1	0	1	1	14	2	1	4	7	1	2	0	0	1
OTU492	3	0	2	11	0	11	3	1	3	5	12	0	6	0	3
OTU98	60	8	212	434	190	85	561	35	2	177	5	119	5	1	2
OTU99	36	113	0	38	4	41	150	132	221	109	6	31	63	40	109
OTU94	50	0	219	0	141	0	0	0	0	0	0	103	0	1	0
OTU95	86	79	37	98	133	22	122	308	98	153	14	130	153	247	178
OTU96	272	0	105	0	127	0	0	0	0	0	0	71	0	0	0
OTU97	18	43	218	1	23	16	21	7	344	15	15	1	39	26	5
OTU90	140	157	133	7	62	41	16	8	303	9	9	37	18	36	1
OTU91	247	35	7	25	2	131	34	40	126	93	36	5	60	65	151
OTU92	34	1	4	11	2	28	1	0	1	0	410	27	0	1	3
OTU93	2	129	0	6	7	1	62	1	2	1	121	7	0	287	8
OTU318	1	0	13	0	3	0	0	0	0	0	0	0	0	0	0
OTU319	15	8	0	29	0	7	1	0	0	2	5	1	13	0	2
OTU310	12	0	13	0	26	0	0	0	0	0	0	21	0	0	0
OTU311	1	5	0	22	0	1	0	5	2	1	3	2	2	1	2
OTU312	2	6	2	2	2	12	7	2	0	12	0	3	20	5	10
OTU313	10	15	12	4	5	47	14	5	5	39	8	5	5	9	7
OTU314	33	0	25	2	0	14	7	6	2	4	43	0	0	0	0
OTU315	0	0	0	0	0	34	7	0	0	20	0	2	0	2	0
OTU316	9	2	2	6	0	4	4	1	17	2	4	2	4	5	1
OTU317	1	22	0	9	0	0	9	8	14	3	1	5	16	7	6
OTU417	1	1	3	4	6	0	2	7	3	2	0	4	0	0	0
OTU416	0	1	0	6	0	0	1	0	0	0	0	4	1	0	1
OTU415	0	13	0	0	0	0	4	0	0	0	0	0	9	3	3
OTU414	0	6	0	0	0	2	1	1	7	0	0	0	0	0	0
OTU413	4	0	2	6	0	4	2	10	0	2	0	1	5	5	1
OTU412	9	0	10	0	7	0	0	0	0	0	0	3	0	0	0
OTU411	1	4	2	2	0	0	2	0	3	2	4	3	4	10	1
OTU410	3	0	0	2	0	0	1	0	0	3	0	1	16	0	9
OTU419	9	6	171	92	1	21	77	4	192	50	1	29	57	24	170
OTU370	0	0	11	0	3	0	0	0	0	0	0	1	0	0	0
OTU309	2	0	13	0	6	0	0	0	0	0	0	0	0	0	0
OTU263	5	0	23	0	10	0	0	0	0	0	0	1	0	0	0

OTU424	78	82	278	98	214	104	145	37	70	193	97	28	37	342	401
OTU389	6	1	0	0	0	0	3	1	0	0	1	0	1	0	0
OTU388	93	0	0	6	0	0	1	2	2	2	5	26	54	95	95
OTU387	1	1	2	2	3	0	1	0	2	0	0	2	0	2	3
OTU386	5	0	1	0	3	2	1	0	1	6	1	3	0	2	1
OTU385	118	79	21	18	23	21	77	11	284	57	88	3	46	9	8
OTU384	1	4	4	5	5	7	1	17	7	1	2	8	7	2	6
OTU383	3	0	36	0	105	54	0	0	0	1	1	131	0	0	0
OTU382	0	6	0	1	0	0	0	0	1	0	1	2	6	1	1
OTU381	1	1	5	2	8	2	2	3	6	4	4	6	6	0	0
OTU380	4	7	0	0	1	0	6	0	5	9	0	1	4	0	2
OTU251	23	2	65	35	5	82	16	26	2	1	38	13	5	4	4
OTU250	36	28	10	11	14	19	3	7	9	10	13	4	0	7	2
OTU253	3	3	0	12	0	469	5	15	14	12	1	110	23	12	13
OTU252	1	0	12	0	46	0	0	0	0	0	0	18	0	0	0
OTU255	3	4	1	42	5	7	13	56	18	10	9	46	1	18	16
OTU254	35	0	0	21	4	3	0	14	2	7	11	30	1	0	1
OTU257	2	1	54	3	9	58	6	1	0	11	22	5	1	0	4
OTU256	2	45	9	0	3	5	0	2	0	1	1	2	21	2	5
OTU259	17	0	7	5	16	15	2	0	2	2	27	2	0	0	0
OTU258	35	0	23	0	14	0	0	0	0	0	0	36	0	0	0
OTU488	3	21	6	10	8	3	22	15	0	1	3	3	5	19	21
OTU426	2	1	6	1	4	1	1	1	0	0	1	1	0	0	0
OTU49	1	539	0	6	1	0	38	10	89	30	0	0	17	54	11
OTU48	462	1	347	0	513	1	0	0	0	0	0	548	0	0	1
OTU47	68	283	24	692	69	349	364	1144	104	293	595	653	387	118	482
OTU46	415	39	121	555	76	17	10	143	569	127	74	26	19	132	12
OTU45	518	6	167	402	350	0	146	143	502	79	85	177	329	296	136
OTU44	0	6	0	6	1	11	514	6	6	1037	25	5	2	2	2
OTU43	54	392	0	289	41	26	374	200	761	326	14	84	610	875	768
OTU42	13	20	259	3	1	1215	0	13	161	1	24	4	0	1	0
OTU41	7	14	4	0	1	0	0	0	744	5	0	1	23	157	5
OTU40	27	0	0	41	1	1132	16	3	0	38	1	42	0	0	1
OTU58	312	8	128	46	549	75	20	18	1	23	8	54	28	17	47
OTU425	2	0	0	0	0	0	0	6	0	0	0	3	1	0	0
OTU59	75	19	228	0	589	0	1	3	151	4	13	147	2	4	0
OTU422	5	0	0	3	1	17	4	1	0	3	0	6	3	0	2
OTU423	0	0	0	4	0	0	2	2	0	0	0	0	5	3	3
OTU174	22	13	1	27	5	51	12	27	6	12	189	8	2	0	1
OTU175	5	0	106	6	41	35	3	2	14	8	147	8	0	1	0
OTU176	53	11	17	38	11	30	28	18	62	74	39	11	25	34	70

OTU177	37	7	54	26	45	102	8	3	6	44	43	13	7	1	4
OTU170	46	19	62	34	40	29	28	1	24	27	31	14	12	91	20
OTU171	127	66	94	89	92	203	58	17	96	84	44	25	64	102	60
OTU172	18	7	6	41	7	1	15	21	14	2	24	51	83	6	28
OTU173	2	23	85	7	114	22	6	7	6	12	5	20	13	21	3
OTU420	0	1	0	5	0	0	0	0	7	0	0	0	0	2	0
OTU178	13	33	12	59	28	13	58	25	24	41	18	52	92	42	71
OTU179	27	1	0	9	0	47	16	6	31	91	11	1	15	86	28
OTU427	0	0	0	0	6	0	0	0	0	0	0	1	0	0	0
OTU421	0	2	1	0	0	0	2	1	2	2	0	4	7	1	1
OTU418	9	0	1	3	6	2	1	7	12	6	1	0	0	9	3
OTU56	112	534	227	142	178	106	74	36	106	144	90	60	66	249	128
OTU57	35	582	31	42	12	47	117	22	20	118	35	9	175	87	39
OTU459	0	0	0	2	0	0	1	0	2	0	0	0	0	0	0
OTU458	0	2	0	1	0	0	0	0	4	0	0	0	0	2	0
OTU453	3	1	0	1	1	0	1	0	2	2	0	1	1	9	3
OTU452	3	2	0	1	1	1	0	3	4	1	2	1	1	0	3
OTU451	0	1	6	0	1	0	1	1	3	1	0	0	0	2	1
OTU450	0	0	0	0	0	1	1	0	1	0	0	0	0	0	0
OTU457	1	2	7	4	15	15	0	73	21	2	0	2	0	0	0
OTU456	0	0	6	4	7	1	4	2	0	0	2	5	1	0	0
OTU455	0	0	3	1	1	2	2	2	6	2	1	3	2	4	3
OTU454	0	0	7	0	4	0	0	0	0	0	0	0	0	0	0
OTU228	8	2	4	9	9	24	8	2	18	29	57	9	8	46	10
OTU229	0	32	0	39	1	8	31	28	14	12	6	56	9	10	3
OTU220	0	0	0	0	0	1	0	0	0	0	59	0	0	0	0
OTU221	0	0	0	0	0	0	4	68	1	24	0	0	0	0	0
OTU222	5	17	0	0	2	0	19	57	21	27	15	1	30	73	52
OTU223	7	0	34	0	42	0	0	0	0	0	0	4	0	0	0
OTU224	0	0	53	0	60	0	0	0	0	0	0	33	0	0	0
OTU225	34	0	103	0	59	0	0	0	0	0	0	47	0	0	0
OTU226	7	0	53	0	42	0	1	0	0	0	0	23	0	0	0
OTU227	20	7	4	4	5	0	3	9	59	4	0	7	37	62	36
OTU358	28	4	51	17	36	18	14	1	13	24	16	16	18	15	7
OTU359	1	3	6	2	0	1	3	0	1	3	1	3	2	3	0
OTU428	0	0	1	0	2	0	0	0	2	0	0	0	0	0	1
OTU429	0	7	0	0	0	0	0	0	0	0	0	0	0	0	0
OTU354	31	5	15	32	7	66	14	4	2	15	57	7	4	6	6
OTU355	5	1	17	4	10	10	2	2	1	4	1	1	2	5	2
OTU356	0	0	0	4	0	0	2	0	2	1	8	8	1	13	11
OTU357	5	2	17	0	10	1	3	3	10	6	26	9	0	1	4

OTU350	16	4	1	8	0	9	3	4	5	5	2	2	6	1	1
OTU351	2	0	11	0	11	0	0	0	0	0	0	7	0	0	0
OTU352	3	0	23	3	5	9	0	0	0	0	6	3	0	0	0
OTU353	10	0	0	10	1	14	6	0	0	4	2	2	4	14	3
OTU129	9	0	122	1	194	0	1	0	0	0	0	81	0	0	0
OTU128	143	21	124	4	176	0	4	1	19	3	3	69	16	4	1
OTU127	92	75	77	129	178	20	94	174	170	30	21	229	92	446	94
OTU126	2	117	0	35	1	2	22	3	34	31	4	33	74	19	11
OTU125	30	91	84	101	77	19	98	160	67	37	6	115	102	240	144
OTU124	52	64	0	19	13	18	142	27	194	76	18	18	83	6	21
OTU123	10	24	21	36	32	11	89	68	20	32	20	50	43	85	61
OTU122	198	27	14	53	33	51	32	57	136	22	5	56	55	77	156
OTU121	33	53	0	146	0	264	19	2	24	120	82	102	3	55	8
OTU120	0	48	0	8	9	375	170	1	0	152	0	0	2	3	4
OTU489	1	0	0	1	0	2	0	0	0	1	1	0	1	0	1
OTU14	153	20	0	377	51	2281	336	79	83	425	1260	80	288	964	466
OTU15	258	138	22	1514	183	1132	1106	2271	89	648	1058	773	844	71	1003
OTU16	51	300	21	439	106	655	665	353	46	209	584	1443	1058	152	753
OTU17	67	502	150	719	529	362	673	1317	399	337	493	1470	559	1635	606
OTU10	1610	121	401	38	64	389	84	15	564	416	298	24	264	856	104
OTU11	412	760	0	1678	2	328	1742	215	183	1322	204	72	8	2	3
OTU12	175	0	1579	0	170	1	0	0	0	1	0	24	1	0	0
OTU13	320	407	376	170	569	74	264	346	519	136	34	444	202	1268	257
OTU18	75	289	21	854	80	220	569	1754	650	329	158	501	454	589	813
OTU19	92	149	168	217	433	104	272	493	267	116	163	1047	308	359	258
OTU462	6	0	23	8	1	16	5	0	27	7	0	4	2	5	5
OTU463	9	6	0	11	0	0	25	25	0	54	151	0	0	3	0
OTU460	3	4	0	0	0	3	1	6	1	0	0	5	0	5	1
OTU461	2	1	8	1	0	1	2	2	0	0	1	2	6	1	1
OTU466	0	2	0	0	0	0	1	3	0	1	2	0	0	0	1
OTU467	1	2	1	2	0	0	2	0	0	0	1	1	1	0	0
OTU464	2	1	1	1	3	0	1	0	0	0	0	1	1	0	6
OTU465	0	0	0	2	0	0	2	0	0	3	7	0	0	0	0
OTU468	0	0	0	0	0	0	0	0	0	0	0	2	0	0	0
OTU469	0	3	0	0	0	0	0	0	0	1	0	0	0	1	0
OTU497	0	1	0	0	2	0	0	2	2	0	0	0	0	2	0
OTU269	175	678	202	97	258	46	237	99	219	59	5	191	105	261	102
OTU495	0	0	0	2	0	1	0	6	1	0	1	2	2	0	4
OTU494	13	25	0	9	4	6	15	1	11	25	3	2	23	5	2
OTU8	0	48	2	914	1	815	217	544	61	114	490	253	1680	4	1591
OTU9	160	37	2072	32	2042	118	12	14	8	45	40	159	7	10	5

OTU6	1512	1795	344	285	312	136	214	230	4	288	36	315	486	10	324
OTU7	120	964	37	1531	129	216	1558	1398	172	851	276	959	1149	117	872
OTU4	198	645	40	969	221	220	1056	1709	920	821	459	436	1348	241	2132
OTU5	329	576	23	528	64	508	754	467	1049	720	540	288	1173	1511	813
OTU2	958	553	928	464	976	361	1100	507	321	821	563	1496	2421	268	1082
OTU3	1503	3725	492	681	62	284	689	147	409	963	1033	165	632	208	873
OTU1	292	2	0	1	0	2	0	2	1	2	7140	8	1	0	1
OTU491	53	0	25	0	121	1	0	0	1	0	21	9	0	3	1
OTU490	0	0	0	0	0	0	0	0	2	0	0	0	0	0	1
OTU307	28	0	21	0	38	0	0	0	0	0	0	38	0	0	0
OTU306	7	5	7	13	7	7	6	7	11	6	5	15	10	1	6
OTU305	1	1	4	4	9	0	0	0	1	3	0	3	0	2	7
OTU304	4	0	0	32	1	7	0	2	3	8	0	0	0	1	0
OTU268	7	27	1	14	3	2	8	4	45	11	2	2	5	17	8
OTU302	10	12	17	6	4	9	4	7	12	10	8	7	11	13	10
OTU301	246	9	1	89	2	199	260	189	118	362	13	183	9	781	45
OTU300	0	10	0	1	0	2	2	0	0	0	1	3	3	3	0
OTU264	6	11	0	4	0	6	11	14	9	12	3	13	23	4	13
OTU265	31	3	17	11	31	17	5	5	8	37	19	4	6	20	21
OTU266	0	0	0	5	0	29	2	0	0	19	3	19	3	3	2
OTU267	11	0	21	0	24	0	0	0	0	0	0	1	0	0	0
OTU260	2	4	15	4	17	7	8	2	10	7	5	4	9	10	5
OTU261	1	46	21	1	12	32	0	2	0	2	7	2	0	1	2
OTU262	20	5	38	18	22	82	21	0	30	38	13	9	7	19	7
OTU308	5	0	20	2	21	1	2	8	0	0	0	21	0	0	1
OTU116	0	0	0	2	0	346	9	1	2	0	0	0	0	1	1
OTU117	131	14	266	81	137	207	103	11	70	135	111	24	69	24	54
OTU114	3	197	0	49	9	6	30	44	9	31	10	15	3	7	7
OTU115	10	33	40	89	27	15	69	67	8	24	16	60	56	75	76
OTU112	55	68	187	49	85	156	40	42	67	75	246	29	17	34	26
OTU113	50	10	134	24	228	50	20	4	1	37	0	21	3	0	2
OTU110	193	89	64	63	87	122	70	27	177	141	84	56	110	110	293
OTU111	68	51	1	390	17	47	340	370	13	162	27	98	87	35	92
OTU118	20	41	0	2	31	7	81	138	34	41	3	4	32	389	41
OTU119	20	0	31	92	1	55	117	12	6	222	4	3	2	7	5
OTU286	2	0	0	0	0	30	6	0	4	12	6	7	29	0	14
OTU287	11	19	13	4	10	9	9	4	23	12	5	2	4	9	15
OTU284	43	23	47	27	9	28	7	1	11	10	41	10	14	39	25
OTU285	7	0	18	0	13	0	0	0	0	0	0	20	0	0	0
OTU282	62	0	52	0	63	0	0	0	0	0	0	69	0	0	0
OTU283	0	0	5	1	4	32	0	4	0	0	0	0	0	0	0

OTU280	11	1	1	14	7	2	22	3	1	3	0	4	1	0	2
OTU281	4	144	0	8	3	9	6	49	71	11	2	6	2	2	4
OTU50	0	0	295	0	472	1	0	0	0	0	0	123	0	0	0
OTU51	364	2	345	0	779	0	1	0	0	0	1	639	0	0	3
OTU52	0	50	1	55	2	1	40	28	578	10	1	5	29	65	2
OTU53	129	130	91	167	172	33	222	346	403	63	84	310	164	139	283
OTU54	259	11	9	174	27	866	227	243	9	204	103	234	753	4	823
OTU55	194	0	559	1	293	0	0	0	0	0	0	36	0	0	0
OTU288	14	15	13	30	13	15	22	30	13	9	9	17	19	28	29
OTU289	65	0	32	3	43	56	0	1	1	1	5	14	0	0	0
OTU163	5	30	20	64	21	24	55	75	15	20	19	66	87	69	104
OTU162	4	135	0	57	0	189	98	3	1	83	1	54	3	0	2
OTU161	119	63	154	17	84	55	28	21	98	36	18	17	18	5	26
OTU160	22	84	10	87	4	1	41	82	16	34	1	22	38	5	5
OTU167	23	126	0	1	1	0	8	5	0	6	0	0	5	0	0
OTU166	156	0	124	0	237	0	1	0	0	0	0	182	0	2	0
OTU165	88	53	34	53	49	61	40	5	86	90	78	28	28	34	29
OTU164	128	16	22	27	46	158	19	16	38	54	54	51	9	47	15
OTU169	9	68	29	1	131	0	15	0	0	18	0	141	1	0	0
OTU168	34	1	166	45	93	270	30	11	1	65	174	81	1	1	0
OTU29	155	0	1008	0	1091	0	0	0	0	0	1	585	0	0	1
OTU28	677	241	2	187	17	1301	361	49	579	1057	23	556	330	2	756
OTU25	116	1746	0	235	15	9	432	565	346	154	2	127	188	330	89
OTU24	301	184	544	504	553	422	596	1013	1	558	556	555	100	5	44
OTU27	24	179	13	21	15	23	64	47	889	50	41	6	69	726	67
OTU26	77	439	747	764	1322	884	374	527	429	116	667	937	587	1134	575
OTU21	120	250	59	589	57	234	550	1230	565	324	171	220	698	517	1001
OTU20	350	81	301	73	277	160	67	18	17	124	22	29	543	124	979
OTU23	1261	7	0	409	10	766	1121	158	1749	2523	870	406	1090	987	731
OTU22	569	4	524	42	960	127	32	3	1	52	68	120	230	135	100

Table S2: OTU network analyses showed 156 core microbiomes in all fecal samples.

OTU ID	Taxonomy	name
OTU507	genus	*Clostridium XlVa*
OTU338	phylum	*Firmicutes*
OTU337	order	*Clostridiales*
OTU349	family	*Lachnospiraceae*
OTU342	order	*Clostridiales*
OTU345	family	*Lachnospiraceae*
OTU159	family	*Lachnospiraceae*
OTU150	order	*Clostridiales*
OTU239	genus	*Oscillibacter*
OTU238	genus	*Flavonifractor*
OTU61	genus	*Parasutterella*
OTU65	family	*Ruminococcaceae*
OTU67	genus	*Oscillibacter*
OTU66	genus	*Bacteroides*
OTU68	order	*Clostridiales*
OTU208	genus	*Anaerotruncus*
OTU206	genus	*Saccharibacteria_genera_incertae_sedis*
OTU204	order	*Clostridiales*
OTU273	family	*Ruminococcaceae*
OTU272	family	*Ruminococcaceae*
OTU105	genus	*Oscillibacter*
OTU104	family	*Porphyromonadaceae*
OTU106	family	*Porphyromonadaceae*
OTU108	family	*Lachnospiraceae*
OTU291	order	*Clostridiales*
OTU290	family	*Lachnospiraceae*
OTU299	family	*Ruminococcaceae*
OTU298	family	*Lachnospiraceae*
OTU198	genus	*Lactobacillus*
OTU192	family	*Ruminococcaceae*
OTU39	genus	*Parabacteroides*
OTU36	family	*Porphyromonadaceae*
OTU34	class	*Deltaproteobacteria*
OTU35	genus	*Clostridium XlVb*
OTU33	order	*Clostridiales*
OTU30	genus	*Alistipes*
OTU31	genus	*Alloprevotella*
OTU83	genus	*Alistipes*

OTU82	family	*Ruminococcaceae*
OTU81	family	*Lachnospiraceae*
OTU80	genus	*Lactobacillus*
OTU87	family	*Lachnospiraceae*
OTU84	family	*Lachnospiraceae*
OTU89	order	*Clostridiales*
OTU328	order	*Clostridiales*
OTU407	family	*Lachnospiraceae*
OTU148	family	*Lachnospiraceae*
OTU140	genus	*Akkermansia*
OTU143	order	*Clostridiales*
OTU144	family	*Porphyromonadaceae*
OTU146	phylum	*Firmicutes*
OTU437	family	*Lachnospiraceae*
OTU436	family	*Lachnospiraceae*
OTU242	genus	*Oscillibacter*
OTU246	order	*Clostridiales*
OTU247	family	*Lachnospiraceae*
OTU72	genus	*Parabacteroides*
OTU73	order	*Clostridiales*
OTU70	family	*Lachnospiraceae*
OTU71	family	*Ruminococcaceae*
OTU77	class	*Clostridia*
OTU74	family	*Desulfovibrionaceae*
OTU75	family	*Lachnospiraceae*
OTU448	family	*Lachnospiraceae*
OTU215	genus	*Parabacteroides*
OTU217	genus	*Butyricimonas*
OTU216	order	*Clostridiales*
OTU210	family	*Lachnospiraceae*
OTU213	family	*Lachnospiraceae*
OTU364	family	*Lachnospiraceae*
OTU130	order	*Clostridiales*
OTU131	genus	*Barnesiella*
OTU132	family	*Lachnospiraceae*
OTU133	phylum	*Firmicutes*
OTU135	genus	*Intestinimonas*
OTU136	genus	*Barnesiella*
OTU138	genus	*Turicibacter*
OTU184	family	*Lachnospiraceae*
OTU180	family	*Lachnospiraceae*

OTU183	family	*Ruminococcaceae*
OTU182	family	*Lachnospiraceae*
OTU98	genus	*Alistipes*
OTU95	family	*Porphyromonadaceae*
OTU97	order	*Clostridiales*
OTU90	kingdom	*Bacteria*
OTU91	genus	*Anaerotruncus*
OTU313	genus	*Acetatifactor*
OTU419	genus	*Clostridium XlVa*
OTU424	family	*Ruminococcaceae*
OTU385	order	*Clostridiales*
OTU384	genus	*Parasutterella*
OTU251	genus	*Clostridium XlVa*
OTU255	order	*Clostridiales*
OTU47	family	*Porphyromonadaceae*
OTU46	family	*Lachnospiraceae*
OTU58	genus	*Roseburia*
OTU176	genus	*Clostridium XlVb*
OTU177	family	*Lachnospiraceae*
OTU170	genus	*Clostridium IV*
OTU171	family	*Lachnospiraceae*
OTU172	kingdom	*Bacteria*
OTU173	order	*Clostridiales*
OTU178	family	*Porphyromonadaceae*
OTU56	genus	*Flavonifractor*
OTU57	order	*Clostridiales*
OTU228	family	*Lachnospiraceae*
OTU358	family	*Lachnospiraceae*
OTU354	genus	*Clostridium IV*
OTU355	genus	*Intestinimonas*
OTU127	genus	*Bacteroides*
OTU125	family	*Porphyromonadaceae*
OTU123	family	*Porphyromonadaceae*
OTU122	genus	*Prevotella*
OTU15	family	*Porphyromonadaceae*
OTU16	genus	*Barnesiella*
OTU17	family	*Porphyromonadaceae*
OTU10	genus	*Mucispirillum*
OTU13	genus	*Prevotella*
OTU18	family	*Porphyromonadaceae*
OTU19	genus	*Bacteroides*

OTU269	genus	*Alistipes*
OTU9	family	*Lachnospiraceae*
OTU6	genus	*Alistipes*
OTU7	family	*Porphyromonadaceae*
OTU4	family	*Porphyromonadaceae*
OTU5	genus	*Odoribacter*
OTU2	order	*Bacteroidales*
OTU3	family	*Lachnospiraceae*
OTU306	order	*Clostridiales*
OTU268	genus	*Anaerovorax*
OTU302	genus	*Arthrobacter*
OTU301	genus	*Clostridium XlVa*
OTU265	genus	*Oscillibacter*
OTU260	family	*Ruminococcaceae*
OTU117	genus	*Butyricicoccus*
OTU115	order	*Bacteroidales*
OTU112	family	*Lachnospiraceae*
OTU110	family	*Ruminococcaceae*
OTU111	family	*Porphyromonadaceae*
OTU287	family	*Ruminococcaceae*
OTU284	family	*Ruminococcaceae*
OTU53	family	*Porphyromonadaceae*
OTU54	family	*Lachnospiraceae*
OTU288	family	*Porphyromonadaceae*
OTU163	family	*Porphyromonadaceae*
OTU161	family	*Lachnospiraceae*
OTU160	family	*Rhodospirillaceae*
OTU165	family	*Ruminococcaceae*
OTU164	family	*Lachnospiraceae*
OTU28	genus	*Acetatifactor*
OTU24	genus	*Alistipes*
OTU27	family	*Ruminococcaceae*
OTU26	family	*Porphyromonadaceae*
OTU21	family	*Porphyromonadaceae*
OTU20	family	*Lachnospiraceae*
OTU22	genus	*Clostridium IV*

Table S3: LEfSe analysis showed 143 specific OTUs which had a significant difference between groups.

	normal_1	normal_2	normal_3	normal_4	normal_5	betaine_1	betaine_2	betaine_3	betaine_4	betaine_5	model_1	model_2	model_3	model_4	model_5
OTU5	0.000754024	0.009441694	0.01530997	0.010785824	0.002098154	0.049536111	0.026653116	0.038455234	0.03439006	0.018883389	0.0166541	0.017703177	0.023604236	0.017309773	0.02471888
OTU11	0	0.002360424	0.007048487	0.013506868	6.56E-05	6.56E-05	9.84E-05	0.000262269	0.00599941	0.024915582	0.010753041	0.006687867	0.043339999	0.055010983	0.057109137
OTU14	0	0.002622693	0.002589909	0.0050159	0.001671967	0.031603449	0.015277186	0.009441694	0.002721044	0.000655673	0.07477953	0.041307412	0.013933056	0.01235944	0.01101531
OTU39	0.01039242	0.019932466	0.012752844	0.009212209	0.025145068	0.0367177	0.006294463	0.007507458	0.054420877	0.01137593	0.000655673	0.001540832	0.003147231	0.002786611	0.002753827
OTU24	0.017834311	0.018194932	0.033209848	0.009867882	0.018129364	0.000163918	0.001442481	0.003278366	3.28E-05	0.006032194	0.013834705	0.018227715	0.018293283	0.016522965	0.019539062
OTU38	0.001803101	0.05140478	0.016326263	6.56E-05	0.000196702	0	0.000163918	0	3.28E-05	0.000229486	0.004688063	9.84E-05	0.038258532	0.031210045	0.036324296
OTU13	0.012326656	0.014555945	0.011343147	0.010490771	0.018653903	0.041569682	0.008425401	0.006622299	0.01701472	0.01334295	0.002425991	0.001114644	0.004458578	0.005573222	0.008654886
OTU43	0	0.002753827	0.006556732	0.001770318	0.00134413	0.028685703	0.025177851	0.019998033	0.024948366	0.012851195	0.000852375	0.000458971	0.010687473	0.009474478	0.012261089
OTU9	0.067927745	0.005212602	0.000458971	0.005245386	0.066944235	0.000327837	0.000163918	0.000229486	0.000262269	0.001212995	0.003868472	0.001311346	0.001475265	0.001049077	0.000393404
OTU32	0	0.00403239	0.016686883	0.001540832	0.001475265	0.017670393	0.024259909	0.016785234	0.011572632	0.01235944	0.000393404	0.001049077	0.005966626	0.012261089	0.014523162
OTU30	0.031210045	0.0166541	0.003245582	0.014949349	0.019244009	0.014818215	0.001311346	0.002524342	0.000688457	0.000163918	0.002819395	0.002393207	0.000426188	0.017145855	0.001311346
OTU29	0.03304593	0.019178441	0	0.005081467	0.035766974	0	3.28E-05	0	0	0	0	3.28E-05	0	0	0
OTU27	0.000426188	0.000196702	0.001540832	0.000786808	0.000491755	0.023800938	0.002196505	0.002262073	0.029144674	0.005868275	0.000754024	0.00134413	0.001639183	0.000688457	0.002098154
OTU82	0.001442481	0.002524342	0.004097958	0.002589909	0.002163722	0.02068649	0.004196309	0.003475068	0.012654493	0.003835688	0.002393207	0.002491558	0.002196505	0.002262073	0.001770318
OTU12	0.0517654	0.000786808	0	0.005737141	0.005573222	0	0	3.28E-05	0	0	3.28E-05	0	3.28E-05	0	0
OTU98	0.006950136	0.003901256	0.001147428	0.00196702	0.006228896	3.28E-05	6.56E-05	0.000163918	6.56E-05	0.000262269	0.002786611	0.000163918	0.005802708	0.014228109	0.018391634
OTU48	0.01137593	0.017965446	0	0.015146051	0.016818018	0	3.28E-05	0	0	3.28E-05	3.28E-05	0	0	0	0
OTU78	0	0.001147428	0.000131135	0.001934236	0.000229486	0	0	0	0	3.28E-05	0.008818805	0.024391044	0.001311346	0.012523358	0.003147231
OTU44	0	0.000163918	0.000196702	0	3.28E-05	6.56E-05	6.56E-05	6.56E-05	0.000196702	0.000196702	0.00036062	0.000819592	0.033996656	0.000196702	0.016850802
OTU57	0.001016293	0.000295053	0.000721241	0.001147428	0.000393404	0.002852178	0.001278563	0.005737141	0.000655673	0.01908009	0.001540832	0.001147428	0.003868472	0.001376914	0.003835688
OTU88	0	0.000163918	0.00062289	0.000262269	0.000229486	0.006491165	0.014785431	0.007474675	0.006327247	0.004360227	0	3.28E-05	0.001475265	0.000393404	0.001212995
OTU40	0	0.001376914	9.84E-05	0.000885159	3.28E-05	0	3.28E-05	0	0	0	0.037111104	3.28E-05	0.001245779	0.00134413	0.000524539
OTU83	0.000163918	0.001835885	0.002721044	0.001081861	0.000458971	0.00429466	0.001639183	0.004556929	0.009835098	0.005737141	0.00036062	0.000196702	0.002589909	0.002163722	0.001934236
OTU75	0.013572435	0.001147428	0.000655673	0.000721241	0.000852375	9.84E-05	0.000163918	9.84E-05	9.84E-05	0.000393404	0.003212799	0.015801724	0.000491755	0.001868669	0.000262269
OTU55	0.018326066	0.001180212	0	0.00636003	0.009605613	0	0	0	0	0	0	0	0	3.28E-05	0
OTU196	0.003343933	0.013146248	0	0.015014917	0.002786611	6.56E-05	0	0	0	0	0	0	0	6.56E-05	0
OTU168	0.005442088	0.002655477	0.00036062	0.001114644	0.00304888	3.28E-05	0	3.28E-05	3.28E-05	3.28E-05	0.008851588	0.005704357	0.002130938	0.001475265	0.00098351
OTU41	0.000131135	3.28E-05	0	0.000229486	3.28E-05	0.005147035	0.000163918	0.000754024	0.024391044	0.000458971	0	0	0.000163918	0	0
OTU73	0.008031997	0.001311346	0.000655673	0.000524539	0.0166541	0.000491755	0.00036062	0.000524539	0.00036062	0.000262269	6.56E-05	0.000131135	0.000262269	0.000590106	0.000819592
OTU103	0.005704357	0.001999803	6.56E-05	0.000491755	0.010195718	0	3.28E-05	0	0	0	0.004917549	3.28E-05	0.001212995	0.001016293	0.000524539

Table S4: LEfSe analysis showed 24 specific species which had a significant difference between groups.

	normal_1	normal_2	normal_3	normal_4	normal_5	betaine_1	betaine_2	betaine_3	betaine_4	betaine_5	model_1	model_2	model_3	model_4	model_5
c__Epsilonproteobacteria	0.070091466	0.00196702	0	0.012097171	0.015178835	0	0	3.28E-05	0	0	3.28E-05	0	3.28E-05	3.28E-05	0
c__Mollicutes	0.009769531	0.00403239	0.000196702	3.28E-05	0.015605022	0	0	0	0	0	0.000163918	6.56E-05	0.002229289	0.00062289	0.000819592
f__Anaeroplasmataceae	0.009769531	0.00403239	0.000196702	3.28E-05	0.015605022	0	0	0	0	0	0.000163918	6.56E-05	0.002229289	0.00062289	0.000819592
f__Bacteroidaceae	0.02973478	0.079893781	0.03806183	0.03609481	0.052027669	0.044487427	0.022522375	0.021735567	0.02471888	0.014621513	0.006687867	0.008458184	0.010884175	0.014949349	0.023112481
f__Clostridiales_Incertae Sedis XIII	3.28E-05	6.56E-05	0.000131135	0.000229486	9.84E-05	0.000557322	0.000262269	0.000163918	0.001475265	0.000885159	6.56E-05	6.56E-05	0.00036062	0.000458971	0.000262269
f__Coriobacteriaceae	0.000262269	0.000163918	9.84E-05	0.000163918	6.56E-05	0.000262269	6.56E-05	0.000163918	0.000131135	0.000131135	0.000426188	0.000262269	0.00036062	0.000655673	0.000524539
f__Helicobacteraceae	0.070091466	0.00196702	0	0.012097171	0.015178835	0	0	3.28E-05	0	0	3.28E-05	0	3.28E-05	3.28E-05	0
f__Lachnospiraceae	0.364357604	0.226797364	0.12788906	0.405599449	0.31514933	0.202963643	0.246893748	0.192407304	0.254663476	0.285742386	0.61498869	0.62134872	0.50250795	0.313149526	0.330623217
f__Oxalobacteraceae	0	6.56E-05	0	0	0	3.28E-05	3.28E-05	0.000196702	3.28E-05	0.000196702	0	3.28E-05	0	3.28E-05	0
g__Anaeroplasma	0.009769531	0.00403239	0.000196702	3.28E-05	0.015605022	0	0	0	0	0	0.000163918	6.56E-05	0.002229289	0.00062289	0.000819592
g__Anaerovorax	3.28E-05	6.56E-05	0.000131135	0.000229486	9.84E-05	0.000557322	0.000262269	0.000163918	0.001475265	0.000885159	6.56E-05	6.56E-05	0.00036062	0.000458971	0.000262269
g__Bacteroides	0.02973478	0.079893781	0.03806183	0.03609481	0.052027669	0.044487427	0.022522375	0.021735567	0.02471888	0.014621513	0.006687867	0.008458184	0.010884175	0.014949349	0.023112481
g__Desulfovibrio	0.001606399	9.84E-05	0	0.00062289	0.001147428	0	0	0	0	0	0	0	0	0	0
g__Dorea	0.001409697	0.000393404	0.000295053	0.002065371	0.005343737	6.56E-05	0	0	9.84E-05	3.28E-05	0.001114644	0.000163918	0.000426188	0.000262269	0.000327837
g__Enterorhabdus	0.000131135	6.56E-05	3.28E-05	0	3.28E-05	0	0	3.28E-05	0	0	0.000163918	9.84E-05	9.84E-05	6.56E-05	6.56E-05
g__Helicobacter	0.070091466	0.00196702	0	0.012097171	0.015178835	0	0	3.28E-05	0	0	3.28E-05	0	3.28E-05	3.28E-05	0
g__Odoribacter	0.017309773	0.014260892	0.01530997	0.018194932	0.017408124	0.049568895	0.0266859	0.038455234	0.03439006	0.018883389	0.016686883	0.017768744	0.023604236	0.017309773	0.024751664
g__Parabacteroides	0.013211815	0.024161558	0.019932466	0.012031603	0.02839065	0.049339409	0.0100318	0.012621709	0.060125234	0.015146051	0.001147428	0.002229289	0.004753631	0.005704357	0.00429466
g__Paraprevotella	0.003999607	0.002655477	0	0.000295053	0.00636003	0	0	0	0	0	0	0	0	3.28E-05	3.28E-05
g__Prevotella	0.012785628	0.01639183	0.013211815	0.016981936	0.019735764	0.044094024	0.013539652	0.008425401	0.021473298	0.014228109	0.004097958	0.001278563	0.005179818	0.007310756	0.009703964
o__Anaeroplasmatales	0.009769531	0.00403239	0.000196702	3.28E-05	0.015605022	0	0	0	0	0	0.000163918	6.56E-05	0.002229289	0.00062289	0.000819592
o__Campylobacterales	0.070091466	0.00196702	0	0.012097171	0.015178835	0	0	3.28E-05	0	0	3.28E-05	0	3.28E-05	3.28E-05	0
o__Coriobacteriales	0.000262269	0.000163918	9.84E-05	0.000163918	6.56E-05	0.000262269	6.56E-05	0.000163918	0.000131135	0.000131135	0.000426188	0.000262269	0.00036062	0.000655673	0.000524539
p__Tenericutes	0.009769531	0.00403239	0.000196702	3.28E-05	0.015605022	0	0	0	0	0	0.000163918	6.56E-05	0.002229289	0.00062289	0.000819592

Table S5: Each phylum microbiome occupied the specific proportion of in each group feces.

tax_name	normal_2	normal_3	normal_4	normal_5	betaine_1	betaine_2	betaine_3	betaine_4	betaine_5	model_1	model_2	model_3	model_4	model_5
Firmicutes	30.91148404	22.14121511	58.16723865	48.82281274	39.52983725	36.57783182	34.42514911	44.63286247	56.51424213	71.84944329	72.22623424	63.54621573	41.66968073	45.09166174
Bacteroidetes	66.39355051	72.83743842	32.23666843	45.37521815	49.61038961	60.12618711	61.67660724	38.43949893	38.08734291	26.09780931	25.78453256	31.45624692	54.28270805	51.58683225
Proteobacteria	1.740704179	4.574712644	3.471488912	3.457473081	7.624527371	2.38243896	2.481299634	14.48893923	4.559472096	0.627319605	0.728728992	2.873091446	2.676487029	1.967934818
Deferribacteres	0.078973346	0.049261084	5.312829989	0.210741216	2.814400789	0.341756761	0.86993772	1.878997868	0.401233544	1.27762998	0.978203782	1.365949762	0.124946569	0.275970826
Actinobacteria	0.210595591	0.295566502	0.16499472	0.072442293	0.197271083	0.338470638	0.306455333	0.176572495	0.175746924	0.082109896	0.177258403	0.318502709	0.927235064	0.676785597
Tenericutes	0.404738401	0.019704433	0.003299894	1.567387797	0	0	0	0	0	0.016421979	0.006565126	0.22328025	0.062473284	0.082134174
Verrucomicrobia	0.157946693	0.026272578	0.353088701	0.138298923	0.101923393	0.095297558	0.14498962	0.309834755	0.208906721	0.003284396	0.003282563	0.137908389	0.197284056	0.200407386
Candidatus Saccharibacteria	0.102007239	0.055829228	0.290390707	0.355625803	0.121650501	0.138017154	0.09556134	0.073294243	0.053055675	0.045981542	0.095194328	0.078804794	0.059185217	0.118273211
Other	0	0	0	0	0	0	0	0	0	0	0	0	0	0

Table S6: Each genus microbiome occupied the specific proportion of in each group feces.

tax_name	normal_1	normal_2	normal_3	normal_4	normal_5	betaine_1	betaine_2	betaine_3	betaine_4	betaine_5	model_1	model_2	model_3	model_4	model_5
Alistipes	26.12283023	22.02166065	22.60223048	19.00519673	24.85762413	6.960667461	21.41412195	24.00248271	5.111638178	36.75879397	16.71249618	17.09208856	11.15358478	30.88740868	20.07273462
Clostridium XlVa	2.615396591	5.316704956	5.310674456	10.78490923	0.304804684	14.35836313	12.78296889	13.17609505	24.66672119	0.716080402	11.10092677	17.37413623	31.09921848	7.2088526	20.38589757
Bacteroides	7.060014011	19.99507713	12.33138609	7.430653979	12.72960616	10.78267779	6.617859551	5.878701898	6.166680298	5.603015075	2.077604644	3.638414892	2.820251444	4.899011603	7.121931508
Barnesiella	1.120884253	14.448638	8.836962294	0.890868597	1.708510468	4.616607072	11.55958	12.66181947	3.099697391	6.444723618	8.432630614	9.462699196	3.975535168	8.594757198	10.31417315
Odoribacter	4.109908928	3.569084345	4.960169942	3.74569751	4.259244405	12.01430274	7.841248435	10.40078028	8.579373518	7.236180905	5.183827274	7.64349175	6.116207951	5.672539751	7.627033034
Parabacteroides	3.136919125	6.046931408	6.457780138	2.47688466	6.946338333	11.95868097	2.9476929	3.413725838	14.99959107	5.804020101	0.356451777	0.958962065	1.231736323	1.869359691	1.323365997
Acetatifactor	0.646065229	4.922874959	1.922464153	5.028008369	0.393037619	0.365514501	7.571524901	3.165454868	5.20160301	3.316582915	16.31530706	3.187138626	10.00679579	3.255264289	4.192342661
Clostridium XlVb	4.288939052	0.943551034	1.030270844	9.482351353	3.007940964	1.040921732	2.2541181	1.028551162	3.01791118	1.582914573	12.67949893	8.574249048	7.849133537	1.708207993	1.636528942
Prevotella	3.035728186	4.102395799	4.280403611	3.495984342	4.828747894	10.68732618	3.978422117	2.278772832	5.35699681	5.452261307	1.273042061	0.549992949	1.342167856	2.395788569	2.99020103
Mucispirillum	3.121351288	0.196914998	0.159320234	10.86589728	0.513355258	6.801748113	1.001830267	2.340840575	4.612742292	1.520100503	3.961706895	4.202510224	3.533809038	0.408250967	0.848570563
Lachnospiracea_incertae_sedis	1.299914377	1.706596652	1.678173128	3.799689546	2.911686853	4.862932062	1.406415567	2.996985281	5.618712685	0.766331658	3.85986353	1.45254548	2.038735984	4.641168887	3.242751793
Alloprevotella	4.172180276	3.142435182	11.25862985	1.113585746	2.21384455	0.039729837	1.261920817	2.358574215	0.44982416	1.256281407	0.468479479	0.564095332	2.514441047	5.296519123	4.96009698
Oscillibacter	3.798552191	0.968165409	0.870950611	2.071944388	3.400978583	5.29996027	4.257778634	0.895548856	2.723480821	2.135678392	2.057235971	3.525595826	2.217125382	1.708207993	0.919284776
Clostridium IV	4.911652526	1.173285199	0.350504514	4.562327057	8.109408839	1.867302344	1.233021867	2.207838269	0.245358633	0.427135678	2.32202872	4.385841207	1.29969419	1.299957026	1.00010102
Parasutterella	0.365844166	1.977354775	9.155602762	0.715394479	0.625651721	1.47794994	3.776129467	3.812732754	1.439437311	1.771356784	0.448110806	1.36793118	2.251104315	4.780833691	1.818365491
Flavonifractor	2.56869308	0.713816869	0.945300053	1.849227239	2.566776289	2.725466826	1.724304017	0.842347934	1.030506257	7.16080402	2.444240758	3.173036243	1.698946653	2.084228621	0.909182746
Helicobacter	16.64201759	0.492287496	0	2.490382669	3.713804444	0	0	0.00886682	0	0	0.010184336	0	0.008494733	0.010743446	0
Lactobacillus	0.326924574	2.166064982	1.030270844	0.526422353	0.296783508	0.476758045	0.443117233	0.576343323	0.114500695	0.615577889	2.403503412	4.56917219	0.399252463	4.437043404	1.080917264
Butyricicoccus	2.12500973	0.213324582	0.116834838	0.998852669	1.227239913	0.190703218	0.5201811	0.611810605	0.588860718	0.175879397	2.260922701	1.946128896	1.163778457	0.923936399	1.060713203
Anaerotruncus	0.171246205	0.073843124	0.509824748	1.822231221	0.080211759	0.619785459	1.550910317	0.585210144	1.210435921	0.502512563	2.250738364	1.861514596	1.129799524	0.526428878	0.60612183
Other	8.359928388	5.808992452	6.192246415	6.843490585	15.30440363	2.852602304	5.856853868	6.756517113	5.765927865	10.75376884	3.381199715	4.470455507	6.150186884	7.39149119	7.889685827

WB original images

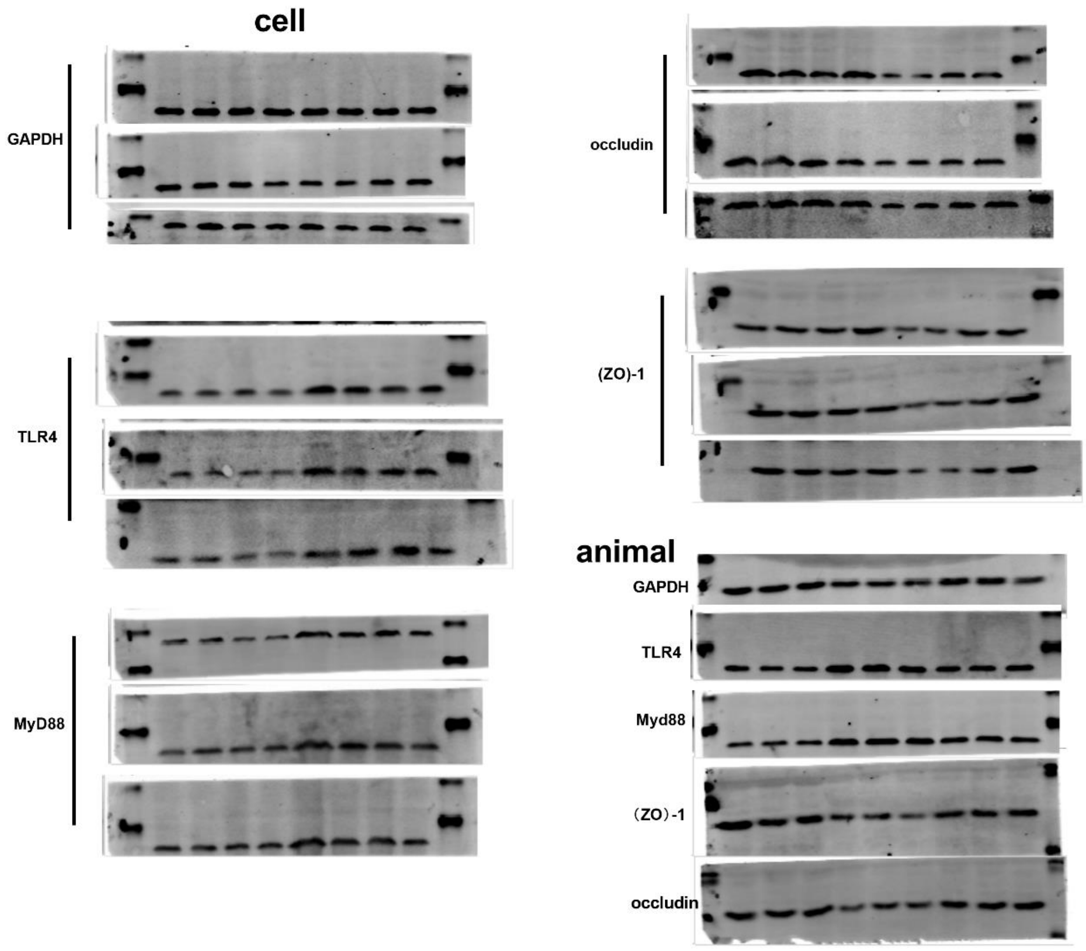

WB original data

**animal**	TLR4	TLR4	TLR4	MyD88	MyD88	MyD88
normal	0.67	0.69	0.65	0.56	0.53	0.58
model	1.32	1.33	1.36	1.57	1.54	1.48
betaine	0.89	0.85	0.84	0.79	0.75	0.77

**animal**	(ZO)-1	(ZO)-1	(ZO)-1	Occludin	Occludin	Occludin
normal	1.54	1.56	1.52	1.32	1.34	1.39
model	0.97	0.91	0.92	0.85	0.82	0.81
betaine	1.34	1.32	1.35	1.09	1.05	1.03

**cell**	TLR4	TLR4	TLR4	MyD88	MyD88	MyD88
normal	0.72	0.73	0.71	0.32	0.28	0.22
betaine only (low)	0.71	0.74	0.69	0.31	0.29	0.25
betaine only (medium)	0.7	0.74	0.68	0.33	0.26	0.28
betaine only (high)	0.73	0.67	0.72	0.31	0.29	0.28
model	1.58	1.62	1.57	0.87	0.91	0.89
betaine (low)	1.32	1.35	1.36	0.67	0.65	0.62
betaine (medium)	1.21	1.23	1.18	0.52	0.51	0.53
betaine (high)	0.98	1.01	0.95	0.41	0.45	0.42

**cell**	TLR4	TLR4	TLR4	MyD88	MyD88	MyD88
normal	0.72	0.73	0.71	0.32	0.28	0.22
betaine only (low)	0.71	0.74	0.69	0.31	0.29	0.25
betaine only (medium)	0.7	0.74	0.68	0.33	0.26	0.28
betaine only (high)	0.73	0.67	0.72	0.31	0.29	0.28
model	1.58	1.62	1.57	0.87	0.91	0.89
betaine (low)	1.32	1.35	1.36	0.67	0.65	0.62
betaine (medium)	1.21	1.23	1.18	0.52	0.51	0.53
betaine (high)	0.98	1.01	0.95	0.41	0.45	0.42
